# Ecogenomics of the SAR11 clade

**DOI:** 10.1111/1462-2920.14896

**Published:** 2019-12-25

**Authors:** Jose M. Haro‐Moreno, Francisco Rodriguez‐Valera, Riccardo Rosselli, Francisco Martinez‐Hernandez, Juan J. Roda‐Garcia, Monica Lluesma Gomez, Oscar Fornas, Manuel Martinez‐Garcia, Mario López‐Pérez

**Affiliations:** ^1^ Evolutionary Genomics Group, División de Microbiología Universidad Miguel Hernández, Apartado 18, San Juan 03550 Alicante Spain; ^2^ Laboratory for Theoretical and Computer Research on Biological Macromolecules and Genomes Moscow Institute of Physics and Technology 141701 Dolgoprudny Russia; ^3^ Department of Marine Microbiology and Biogeochemistry Royal Netherlands Institute for Sea Research (NIOZ) Texel The Netherlands; ^4^ Department of Physiology, Genetics, and Microbiology University of Alicante Alicante Spain; ^5^ Flow Cytometry Unit, Pompeu Fabra University (UPF) and Centre for Genomic Regulation (CRG) The Barcelona Institute for Sciences and Technology (BIST) Barcelona Spain

## Abstract

Members of the SAR11 clade, despite their high abundance, are often poorly represented by metagenome‐assembled genomes. This fact has hampered our knowledge about their ecology and genetic diversity. Here we examined 175 SAR11 genomes, including 47 new single‐amplified genomes. The presence of the first genomes associated with subclade IV suggests that, in the same way as subclade V, they might be outside the proposed Pelagibacterales order. An expanded phylogenomic classification together with patterns of metagenomic recruitment at a global scale have allowed us to define new ecogenomic units of classification (genomospecies), appearing at different, and sometimes restricted, metagenomic data sets. We detected greater microdiversity across the water column at a single location than in samples collected from similar depth across the global ocean, suggesting little influence of biogeography. In addition, pangenome analysis revealed that the flexible genome was essential to shape genomospecies distribution. In one genomospecies preferentially found within the Mediterranean, a set of genes involved in phosphonate utilization was detected. While another, with a more cosmopolitan distribution, was unique in having an aerobic purine degradation pathway. Together, these results provide a glimpse of the enormous genomic diversity within this clade at a finer resolution than the currently defined clades.

## Introduction

The SAR11 clade, as originally defined by 16S rRNA sequences (Giovannoni *et al*., 1990), is a group of extremely successful pelagic bacteria, among the most abundant in aquatic environments, accounting for about 25% of the plankton cells in upper regions of the ocean photic zone (Morris *et al*., 2002; Rappé *et al*., 2002; Thorpe *et al*., 2007; Salcher *et al*., 2011). This clade has been characterized as composed of photoheterotrophic microbes with streamlined genomes and high surface‐to‐volume ratio that, together with the ability to oxidize a wide variety of one‐carbon compounds and the use of light by proteorhodopsin, are particularly well suited for the oligotrophic conditions of most aquatic environments (Giovannoni, 2017). Using 16S rRNA gene and the internal transcribed spacer (ITS), members of SAR11 have been divided into diverse lineages (or clades) with different spatiotemporal abundance patterns (Carlson *et al*., 2009; Brown *et al*., 2012; Morris *et al*., 2012; Vergin *et al*., 2013; Thrash *et al*., 2014). Due to their importance in nutrient fluxes through marine food webs (Giovannoni, 2017), these microbes are among the most relevant for ecosystem functioning in the oceans. The amount of genomic information on these microbes increases enormously with each metagenomic study of marine waters because of their widespread distribution and abundance throughout the surface oceans (Giovannoni, 2017).

Although the number of strains has increased notably in the last few years (Rappé *et al*., 2002; Giovannoni *et al*., 2005; Stingl *et al*., 2007; Oh *et al*., 2011; Grote *et al*., 2012; Jimenez‐Infante *et al*., 2017), in‐depth analysis has been hampered by the difficulty in obtaining large numbers of pure cultures using standard methods and carrying out experiments with such slow‐growing microbes. The problem has been compounded by the poor assembly output obtained from most metagenomes and the scarcity and low reliability of metagenome‐assembled genomes (MAGs) (Tully *et al*., 2017). Despite the abundance of SAR11‐related reads found in most metagenomes, the yield of contigs obtained is low, likely due to the high genomic diversity within populations that, in addition, are subjected to frequent recombination (Vergin *et al*., 2007). This has limited the number of genomes available and has prevented the application of the powerful analytical approaches of genomics to understand the relationship between genome diversity and metabolic potential with environmental conditions. These problems could be solved with long‐range sequencing, but the error rate of these systems precludes their intensive use in metagenomics yet (Sedlazeck *et al*., 2018).

Single‐cell genomics (SCGs) has been shown to be another alternative method to retrieve genomes of microorganisms that are currently difficult to culture (Lasken, 2012). This approach overcomes some of the metagenomic limitations by assembling individual genomes (single‐amplified genomes, SAGs), one at a time, albeit, it generates most often incomplete genomes. Some SAR11 studies have already used this technology (i) for the evolutionary analysis of the marine‐to‐freshwater transition of this clade (Zaremba‐Niedzwiedzka *et al*., 2013), (ii) to suggest the important contribution of SAR11 to nitrite production in oxygen minimum zones (Tsementzi *et al*., 2016) as well as (iii) to describe the first bathypelagic specific SAR11 representatives (Thrash *et al*., 2014).

Here, we took advantage of the combination of large metagenomic data sets available (Thrash *et al*., 2014; Luo *et al*., 2015; Berube *et al*., 2018; Henson *et al*., 2018) with all the pure culture genomes, MAGs and SAGs (including 47 obtained for this work from a single sample at a Mediterranean Sea station) and used phylogenomics combined with metagenomic read recruitment to expand SAR11 classification, including the new genomes. We found a significant overlap between the phylogenomic classification and metagenomic distribution patterns that we have called genomospecies. They were more abundant in certain latitudes, temperatures, seasons, depths and/or availability of nutrients that sometimes were linked to the genomospecies‐specific gene content. In addition, within genomospecies, we detected that genomic microdiversity across the water column at a single location was higher than in surface waters at different locations across the global ocean. Our work demonstrates the power of using a population genomics approach (a combination of SCGs, metagenomics and environmental distribution data) to provide a more representative picture of the metapopulations of SAR11.

## Results and discussion

### *Genomic classification of the SAR11 clade*

We collected a total of 175 SAR11 genomes from publicly available databases (largely pure cultures and SAGs, together with a few MAGs) (Thrash *et al*., 2014; Luo *et al*., 2015; Tsementzi *et al*., 2016; Berube *et al*., 2018; Henson *et al*., 2018; Tully *et al*., 2018). We added 47 manually curated SAGs obtained from a single sample in the Mediterranean Sea (Blanes Bay Microbial Observatory) (Table S1). SAGs named from SAG‐MED01 to SAG‐MED15 were amplified using the new thermostable Equiphi29 polymerase (Stepanauskas *et al*., 2017). They showed higher completeness (mean 74 ± 18%) than SAGs amplified with the phi29 DNA polymerase (mean 66 ± 17%). Despite the fact that several other studies have provided many other SAR11 representatives (Hugerth *et al*., 2015; Luo *et al*., 2015; Tsementzi *et al*., 2016; Tully *et al*., 2018), we have only included in our study genomes with > 50% completeness and < 5% contamination (Table S2).

**Figure 1:**
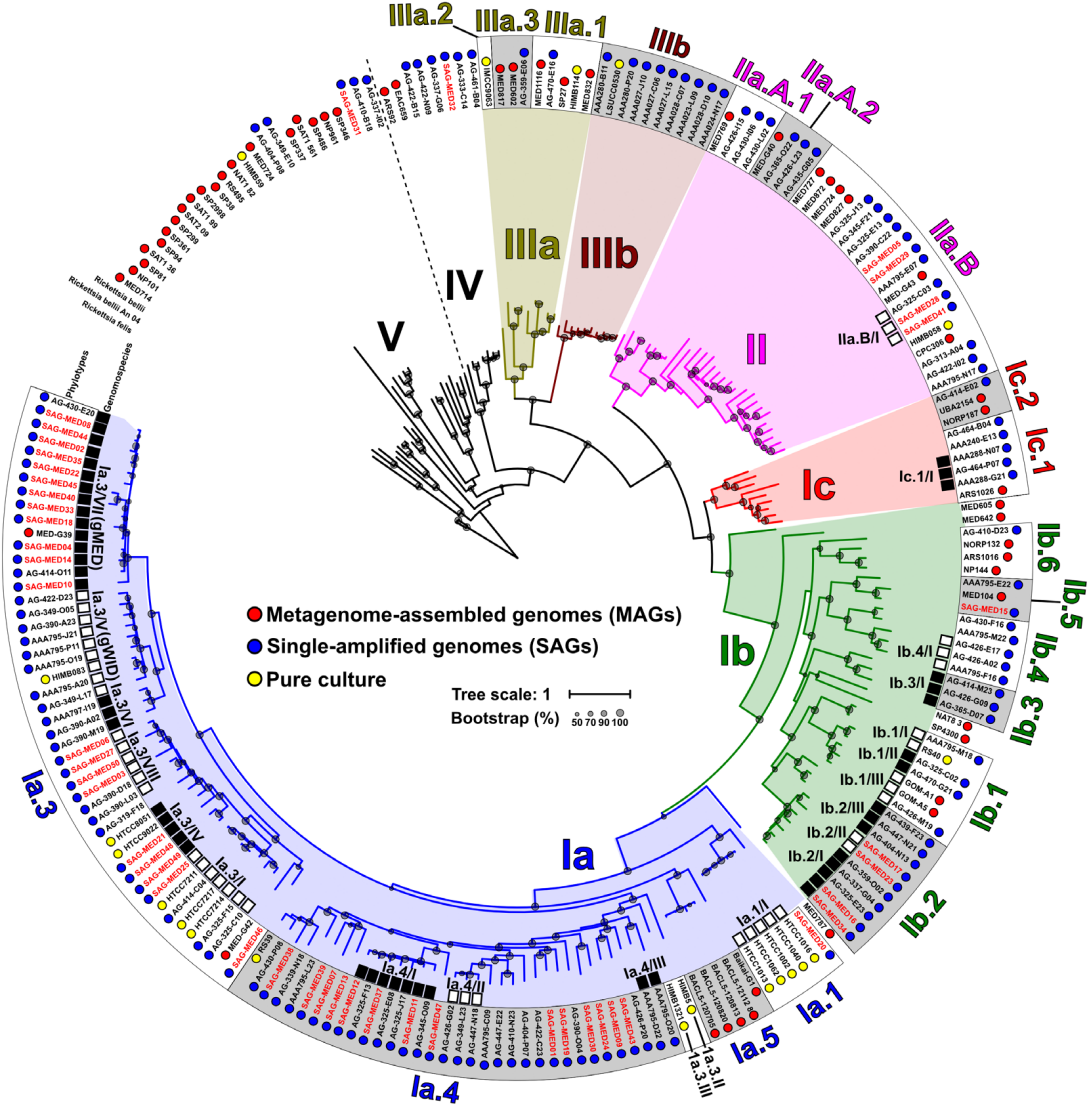
Maximum likelihood phylogenomic tree of all SAR11 genomes available to date, together with those retrieved in this study (SAG‐MED, highlighted in red). Coloured dots next to the genome identifier indicate the origin of the genome, that is MAG (red), SAG (blue) or pure culture (yellow). Branches of the tree were coloured according to the previous classification (Giovannoni, 2017). Sequences were grouped within Subclades and genomospecies (black or white squares). Only subclades with at least 3 SAGs or pure culture genomes are shown.

The phylogenomic tree in Fig. 1 shows the clusters formed by a concatenation of 232 genes present in all genomes (#221). A comparison of this classification with the phylogenetic reconstruction based on rRNA ITS (Fig. S1) showed that both trees have almost the same topology, with the exception of subclade Ia.2, which appeared as part of phylotype Ia.3 in the phylogenomic classification (Fig. 1), as it has also been recently suggested (Delmont *et al*., 2019). We have found a remarkable degree of diversity despite the relative conservation of the 16S rRNA sequences or the overall synteny. This increased diversity has allowed us to tentatively discriminate new subclades following the previous nomenclature (García‐Martínez and Rodríguez‐Valera, 2000; Giovannoni, 2017; Delmont *et al*., 2019) (see Experimental procedures section). A total of 21 subclades were discernible. As shown in Fig. 1, they are overlapped over the extant 16S rRNA classification (Vergin *et al*., 2013; Giovannoni, 2017). Half of these subclades (#10) have no cultured representatives.

Genome pairwise comparison showed that the minimum average nucleotide identity (ANI) value was consistent within the subclades at *ca*. 75% (average amino acid identity, AAI, 70%) (Table S3 and Fig. S2), similar to the cut‐off accepted to designate members of different genera (Konstantinidis and Tiedje, 2005). Subclade Ia was the most represented group, with 95 genomes. The majority of these sequences affiliated with the previously identified phylotypes Ia.1, Ia.2 and RS1‐4 (Ngugi and Stingl, 2012) (denoted here Ia.4 for simplicity). We have added phylotype Ia.5 which comprised five MAGs, four of which were collected from the Baltic Sea (a brackish environment) (Hugerth *et al*., 2015) and one from Lake Baikal (freshwater) (Cabello‐Yeves *et al*., 2018) (Fig. 1). Although the pure culture genomes (HIMB5 and HIMB1321) have recently been classified as groups within subclade 1a.3 (1a.3.II and Ia.3.III) (Delmont *et al*., 2019), our data (Fig. S2) and the resulting phylogeny (Fig. 1) show them as an outgroup of Ia.4. Our phylogenomic analyses (Fig. 1 and ITS, Fig. S1) also expanded the number of phylotypes within the other SAR11 subclades. For instance, subclade Ib was further divided into six novel phylotypes.

We have also found the first genomes associated with the ITS‐defined subclade IV (Thrash *et al*., 2014). This group like subclade V have greater genomic divergence at nucleotide and protein level (ANI, AAI and synteny) (Figs 1 and S3) and have the 16S, 23S and 5S rRNA ribosomal genes forming a single operon rather than split by the lipopolysaccharide‐O‐chain synthesis genomic island, as is the case of all other *bona fide* Pelagibacterales. Therefore it is controversial if they belong to this order (Viklund *et al*., 2013) and have been not studied further. The same applies to subclades that appeared as outgroups containing only one MAG as a representative, due to the high risk of chimerism.

### *Patterns of metagenomic recruitment*

We analysed the distribution patterns along a metagenomic data set consisting of 620 metagenomes, including 140 worldwide samples from the *Tara Oceans* Project (Sunagawa *et al*., 2015), 480 samples from GEOTRACES cruises collected from diverse regions of the Atlantic and Pacific Oceans (Biller *et al*., 2018) and a depth profile in the Mediterranean Sea (Haro‐Moreno *et al*., 2018). For metagenomics recruitment, we used only subclades with at least three SAGs or pure cultures and performed the recruitment for all of them with a high identity threshold (≥98%). A threshold of at least three reads recruited per kilobase of genome per gigabase (RPKGs) of metagenome and a coverage of > 70% were used to establish the presence of the genomes in a metagenomic sample (Table S4). In order to avoid unspecific recruitment, we also removed the complete ribosomal operon from all the genomes. Previous studies highlighted the high similarity of the 16S rRNA gene throughout the SAR11 clade, for example, members of the Ia subclade share 16S rRNA gene identities higher than 98% while the genomes have AAIs lower than 80% (Grote *et al*., 2012). Furthermore, it has been suggested that the ribosomal operon in SAR11 suffers frequent homologous recombination due to the exchange of different versions of the O‐chain cluster (Wilhelm *et al*., 2007; López‐Pérez *et al*., 2014) what would make it an unreliable marker for close relatives. Fig. S4 shows the effect of the removal of the ribosomal operon over the measured in RPKGs of SAR11 in different metagenomes. It significantly increased the RPKG values, very often with values three times higher.

**Figure 2:**
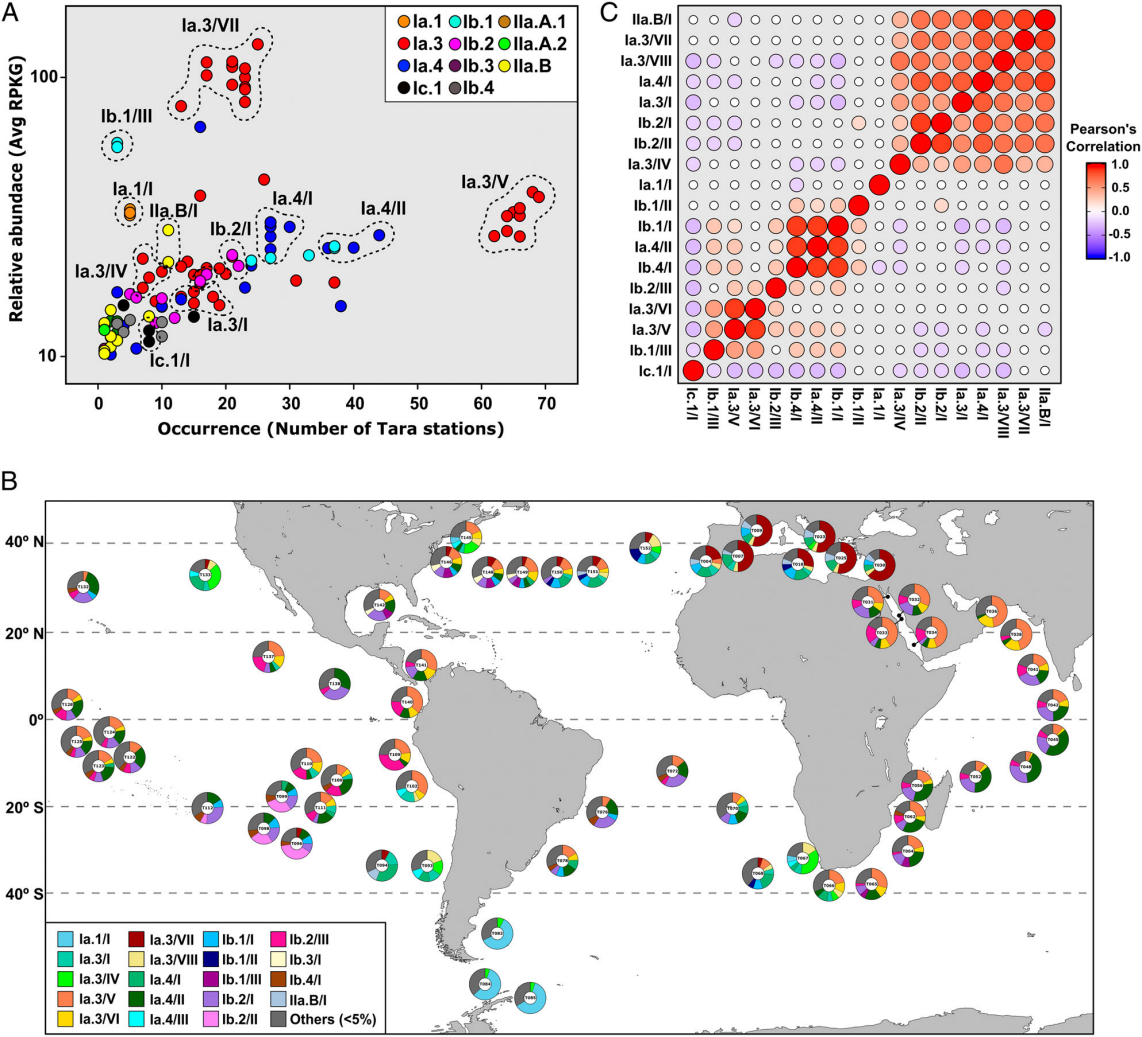
A. Occurrence plot of SAR11 genomes within *Tara* stations. The horizontal axis stands for the number of metagenomic samples one genome recruited at least three RPKG (presence), while the *y*‐axis represents the average relative abundance (RPKG, semi‐log scale) of one genome within the samples where it is present. Genomes are coloured according to their subclade. Dashed lines correspond to delimited genomospecies. B. Relative abundance of SAR11 genomospecies in surface *Tara* Ocean metagenomes. For each sample, those genomospecies with less than 5% of relative abundance are included in ‘Others’. C. Linear Pearson's correlation coefficients between SAR11 genomospecies abundances. Only comparisons with *p*‐value ≤ 0.05 are shown.

Analysis of the relative abundance throughout *Tara* stations revealed that, within the same phylogenomic subclade (clustered together in the phylogenomic tree of Fig. 1), there were groups of genomes that recruited consistently along sampling sites (Fig. 2A and Table S4) and they were remarkably consistent in both *Tara* and GEOTRACES data sets (Figs S5 and S6). We used these similar recruitment patterns to group the genomes into smaller units (genomospecies). In the end, we were able to differentiate 20 genomospecies within nine phylogenomic subclades (Fig. 1). The minimum pairwise ANI value within the genomospecies was *ca*. 80% (Table S3). Whether genomospecies can be considered taxonomic units is an issue that we would rather not tackle, but it seems clear that, in the future, genomic and ecological standards will slowly replace or complement the more classical phenotype‐derived criteria.

### *Distribution of genomospecies across latitudes, depth and seasonal profiles*

We analysed the global distribution of SAR11 genomospecies along the *Tara Oceans* transect (Sunagawa *et al*., 2015) (Table S4). Some genomospecies were more abundant in specific regions such as the Mediterranean Sea (Ia.3/VII and IIA.B/I) or the South Pacific (Ib.1/II) (Fig. 2B). In the same way, genomospecies corresponding to previously described subclades, such as Ia.1 and Ic.1 were limited to the Southern Ocean (Fig. 2B) and deep waters worldwide respectively (Brown *et al*., 2012; Thrash *et al*., 2014). However, other genomospecies presented a widespread distribution in temperate (Ia.3/VIII, Ia.3/I, Ia.4/I and Ib.2/I) or tropical waters (Ib.1/I and Ib.2/II), while representatives of Ia.3/V, Ia.3/VI and Ib.1/III were found in several ocean provinces from 40°N to 40°S (Fig. 2B).

One major exception was genomospecies Ia.3/VII that, not surprisingly, was mostly represented in the Mediterranean (from where 47 of the analysed SAGs were obtained). This group showed the highest recruitment values of any genomospecies at any station (Fig. 2A and Table S4). Ia.3/V is also anomalous in being the most widespread genomospecies but with smaller average abundance (Fig. 2A and Table S4). Hereafter, these two genomospecies within clade Ia.3 will be referred to as Mediterranean (gMED) and widespread (gWID) genomospecies. These distribution patterns are also consistent within a co‐occurrence plot (Fig. 2C) emerged from the *Tara* metagenomic recruitments.

The *Tara* data set was the largest available at the time but covers mainly subtropical latitudes and has mostly surface or subsurface samples. Other smaller data sets, such as those from the Mediterranean Sea described by Haro‐Moreno and colleagues (2018), allowed us detecting differential recruitment of the genomospecies at different depths at the same location, while others such as GEOTRACES provided latitudinal gradients, or seasonal variations in the BATS and HOT stations (Biller *et al*., 2018).

#### *Depth profile*

To investigate the vertical distribution throughout the water column, we used the recruitment of metagenomic reads from a metagenomic profile in a stratified Western Mediterranean water column, as well as during the winter when the water column was mixed (Haro‐Moreno *et al*., 2018). Among the genomospecies recruiting at this site, Ia.3/VI, Ia.4/II and Ib.2/I were confined to surface waters (stenobathic), Ia.4/I appeared at several depths within the photic zone (eurybathic) (Fig. S7 and Table S4). However, all of them presented their maxima at 15 m except for members of Ia.3/VIII that reached their maximum at the deep chlorophyll maximum (DCM) and during the mixing period at all photic depths, suggesting a better adaptation to nutrient‐rich environments. Although less abundant than during the stratified period, gMED, Ia.3/VIII, Ia.3/I, Ia.1/IV, Ia.4/I, Ib.2/I, Ib.2/II and IIa.B/I were also present throughout the winter mixed water column (Fig. S7 and Table S4). As already described (Thrash *et al*., 2014), Ic.1/I was more prevalent in mesopelagic or bathypelagic depths and was not found in the photic zone.

#### *Seasonal variation*

We have used a 2‐year metagenomic time series collected at monthly intervals at HOT and BATS stations during the GEOTRACES cruises (Biller *et al*., 2018). In the BATS station, which is also subjected to winter mixing events, we found that four genomospecies designated above as particularly abundant in the Mediterranean Sea and other temperate waters (gMED, Ia.3/VIII, Ia.4/I and Ib.2/I) and another with a more widespread distribution (Ib.1/III) recruited enough to follow their dynamics throughout yearly cycles (Fig. S8, Table S4). While the gMED tended to peak during spring–summer stratification and decrease during winter mixing, Ib.1/III abundance seemed to take advantage of the disturbances introduced by the upwelling of nutrients and/or subsequent phytoplankton blooms (Haro‐Moreno *et al*., 2018) (Fig. S8A).

To refine this picture, we examined the recruitment of SAG AG‐430‐E20, a representative of gMED within three specific BATS metagenomic samples (labelled 1, 2 and 3 in Fig. S8B) collected during two periods of strong stratification (1 and 3) and the mixing event in between. We observed that during stratification, most of the aligned reads recruited at nucleotide identities greater than 98% (Fig. S8B). However, during mixing, the recruitment pattern changed and most of the reads were recruited at 95% to 85% identity. These results suggest that another unknown genomospecies, which is better adapted to the new environmental conditions during the winter, replaced the clonal frame represented by SAG AG‐430‐E20. Then, for the following stratified period, the same gMED population of SAG AG‐430‐E20 (with the genomic islands at the same location) was recovered. Apart from the genomic island related to the cell wall glycosylation, the large number of hypothetical proteins made it impossible to infer the function of the rest of the islands. Therefore, genomospecies vary with depth within the photic zone when the water column is stratified or between seasons, allowing the coexistence of physiologically distinct lineages. However, the intense winter convection produces the competitive exclusion of all but a few genomospecies.

In the permanently stratified HOT station, Ia.4/II and Ib.1/III were the most abundant (Fig. S8C). Despite permanent stratification, spring and summer conditions seemed to be more favourable for Ia.4/II and Ib.1/III, allowing them to reach depths down to 100 m (Fig. S8C).

#### *Latitudinal gradients*

**Figure 3:**
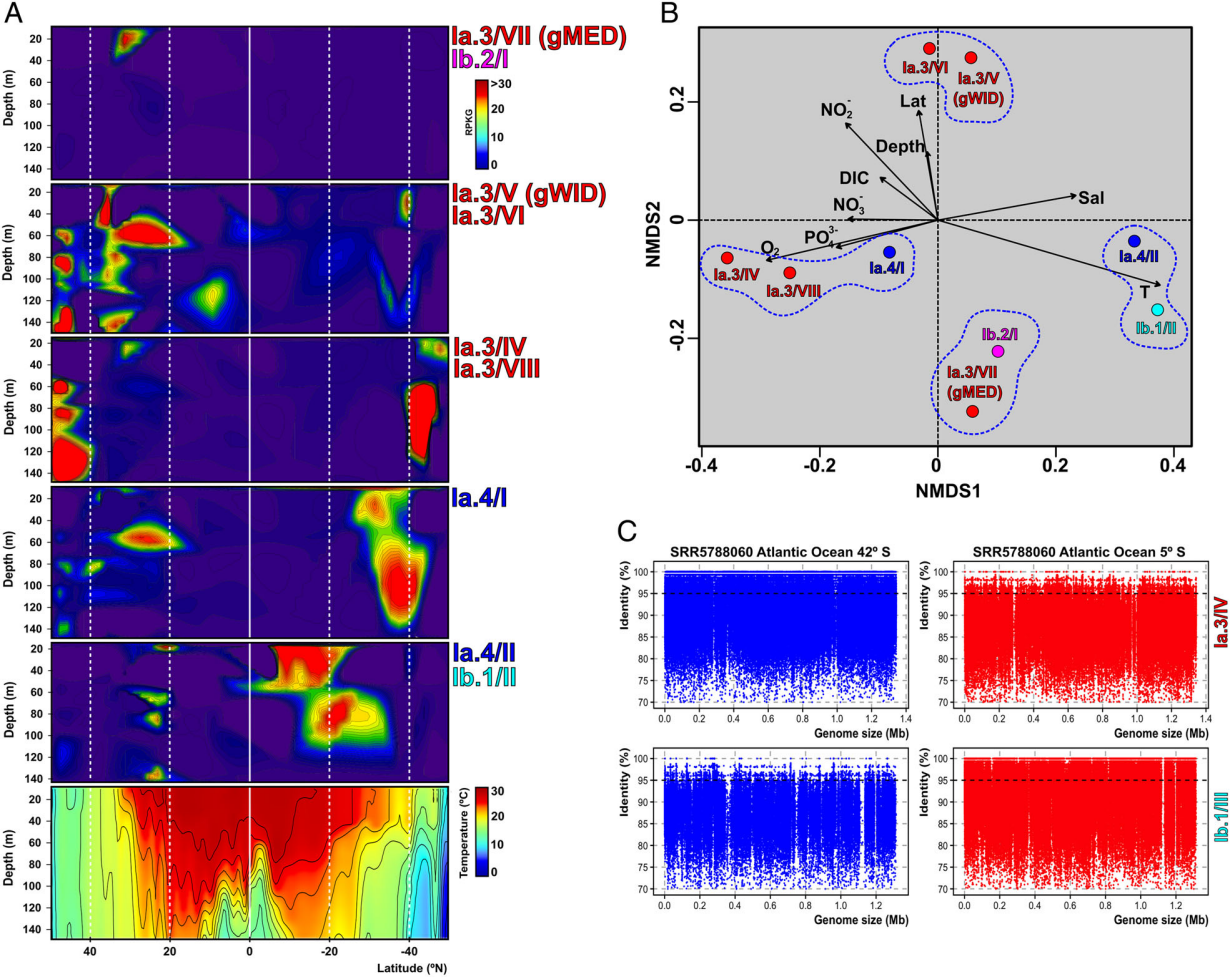
A. Latitudinal transect following the GEOTRACES GA02 cruise. The first five boxes show the recruitment of different genomospecies by depth and latitude. Genomospecies that showed the same distribution were combined in the same figure. The last box shows the temperature profile. B. NMDS analysis of genomospecies according to Bray‐Curtis distance between GEOTRACES GA02 cruise samples. Only fitting statistically significant (*p*‐value ≤ 0.05) physicochemical parameters are shown. NMDS stress value: 0.116. C. Recruitment plots of one representative genome of IA.1/IV (found in cold latitudes) and IB.1/III (found in warm latitudes) in two metagenomes.

To determine the distribution and abundance in a latitudinal gradient, we analysed the recruitment of genomospecies along 127 samples in a transect of 32 stations from 50°N to 50°S in the West Atlantic Ocean (GEOTRACES GA02 cruise (Biller *et al*., 2018)). We found a shift of different genomospecies peaking at specific latitude ranges (Fig. 3A). Furthermore, their correlation with environmental parameters showed a clustering in four groups (Fig. 3B): (i) Ia.4/II and Ib.1/II were dominant in tropical latitudes (warm and oligotrophic), particularly in the southern hemisphere; (ii) Ia.3/IV, Ia.3/VIII and Ia.4/I were the most prevalent at higher latitudes above 40°N and 40°S. These three genomospecies were correlated with an increase of inorganic nutrients (such as ${\mathrm{NO}}_{3}^{-}$ and ${\mathrm{PO}}_{4}^{3-}$) typical of colder or deeper waters; (iii) gWID and Ia.3/VI were found only in northern latitudes higher than 20° and (iv) gMED and Ib.2/I were only detected in a few samples of this transect, all close to the BATS station, possibly taking advantage of the special features of the central North Atlantic gyre that is often considered to be a P‐limited environment (Fig. 3B) (see below). Figure 3C shows the recruitment plots of the two genomes belonging to the two more opposite trends regarding latitudinal preferences, the ‘cold’ Ia.3/IV and the ‘warm’ Ib.1/III, in two samples, one near the equator (5°S) and another at 42°S (Fig. 3A). In both examples, in the recruitment pattern at the less favourable environment, reads at higher than 95% identity disappeared and metagenomic islands appeared (mostly hypothetical proteins) that may be related to the adaptation to the specific habitat (Fig. 3C). In polar latitudes no transect was available, but in the *Tara* samples from the Southern Ocean near the Antarctic Peninsula, the dominant genomospecies was Ia.1/I, mostly represented by pure cultures from coastal Oregon (Brown *et al*., 2012) (Fig. S6).

These results highlight how SAR11 is actually formed by a complex mixture of populations adapted to different environmental ranges as previously noted (Morris *et al*., 2002; Carlson *et al*., 2009; Giovannoni and Vergin, 2012; Thrash *et al*., 2014). For example, subclade Ia.1 has been associated with cold waters and subclade Ia.3 with warm waters (Giovannoni, 2017). However, within subclade Ia.3 we can now differentiate several groups. While gMED was only found in warm waters with low P levels, gWID, and to a lesser extent Ia.3/VI were cosmopolitan. Ia.3/VIII was restricted to mixed and nutrient rich waters, and Ia.3/IV and Ia.3/I were preferentially found in temperate waters.

### *Comparative genomics of gMED and gWID*

The existence of a biogeography in microbes, despite recent evidence (López‐Pérez *et al*., 2013; Swan *et al*., 2013), and that provided by this study is still a controversial issue. From Fig. 1, it is easy to conclude that several of the SAR11 subclades coexist in the same environment, since SAGs retrieved from the same sample in a single location in the Mediterranean Sea had representatives in all the subclades (with the obvious exceptions of freshwater dwellers Ia.5 and IIIb (*Fonsibacter*) or the deep ocean clade Ic). However, although our results showed no evidence of a significant biogeography, we detected the apparent endemism of a few genomospecies, like gMED in the Mediterranean Sea, that could correlate their distribution with the presence of some specific metabolic traits encoded in these genomes but absent in other genomospecies.

To analyse the genomic differences that could reflect ecological adaptations, we focused on phylotype Ia.3 that was the largest with 47 genomes and could be split into six genomospecies, providing the two with the most different distribution patterns (Fig. 2A). While gWID could be considered the most cosmopolitan, present in *ca*. 56% of *Tara* samples (75% if only surface and DCM samples were considered, Table S4), gMED was restricted to Mediterranean Sea and BATS station (North Atlantic central gyre) metagenomes (Figs. 2A,B, S5 and S6 and Table S4).

**Figure 4:**
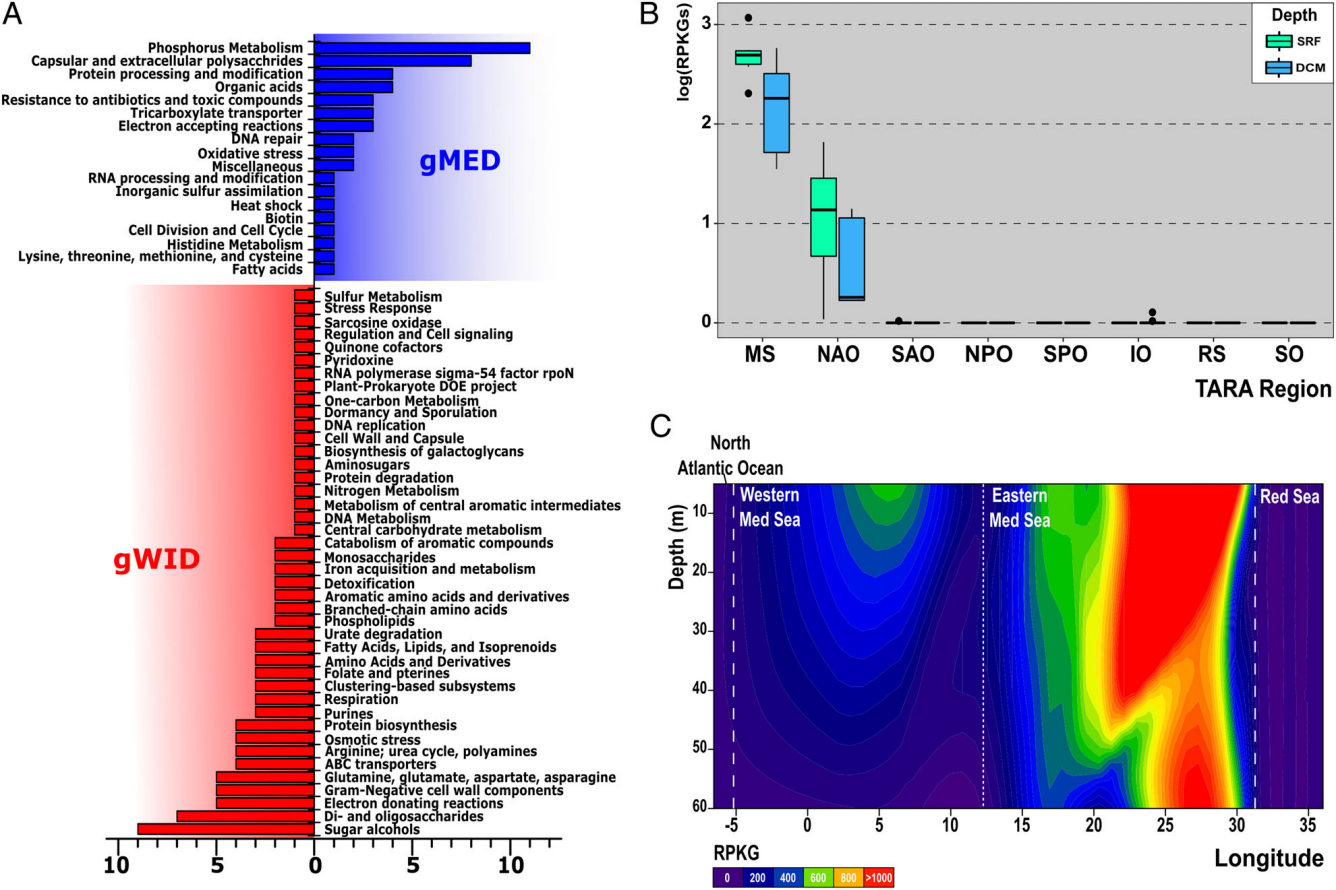
A. Pangenome analysis between genomes of genomospecies gMED and gWID. Functional characterization of the pangenome using SEED Subsystems database for the number of differential genes between genomospecies gMED and gWID. B. Boxplot indicating the recruitment values (*x*‐axis, log scale) of the phosphonate cluster at two depths [surface (SRF) and DCM] among *Tara* regions: IO, Indian Ocean; MS, Mediterranean Sea; NAO and SAO; North and South Atlantic Ocean; NPO and SPO, North and South Pacific Ocean; RS, Red Sea and SO, Southern Ocean. C. Longitudinal transect of the Mediterranean Sea showing the recruitment values of the phosphonate cluster.

We sought differences in their overall gene content through pangenome analysis (see Experimental procedures section). The common part of both pangenomes was excluded from being able to analyse only the specific adaptive components of each at the functional level using the SEED Subsystems database (Overbeek *et al*., 2005). Leaving aside categories related to the production of the skeletons of sugars and glycoproteins that decorate the surface of the cell, contained in the HVR2 hypervariable region (Grote *et al*., 2012), we identified an increased proportion of genes related to ‘Phosphorus Metabolism’ in gMED (Fig. 4A), mainly due to the presence of the complete cluster for the transport and utilization of phosphonates (Pn). The Pn cluster of SAR11 was previously described in the HTCC7211 genome (Ia.3/I), which was able to grow using Pns as a source of phosphate in laboratory experiments (Carini *et al*., 2014). This cluster is located in a flexible region of the genome (Fig. S9). Both genomospecies shared the complete gene cluster for phosphate acquisition (pstSCAB‐phoU) and their regulation (PhoB‐PhoR) located at the same position within the same flexible genomic island, although they had less than 60% amino acid identity (Fig. S9). However, while both gMED and gWID had the first three genes of the Pn operon *phnDCE*, coding for the Pn ABC transporter located at equivalent loci, the complete cluster (*phnF‐phnN*) required for the catalytic activity was only found in genomes of gMED (Fig. S9). Remarkably similar to what has been previously found in *Prochlorococcus* (Feingersch *et al*., 2012), the shared part of the gene cluster for Pn‐specific ABC transporter of both genomospecies differs not only in sequence (only *ca*. 30% amino acid identity) but also in gene order (*phnDCE* or *phnCDE*) (Fig. S9). In the case of *Prochlorococcus*, growth assays showed that only strains with the *phnCDE* format and containing the rest of the Pn cluster were able to grow on phosphite and phosphonate (Feingersch *et al*., 2012). These similarities between cyanobacteria and SAR11 could suggest either a common origin by horizontal gene transfer of the gMED Pn cluster (from a similar donor) or convergent evolution of picocyanobacteria and Pelagibacterales driven by the scarcity of phosphate.

To gain more insights into the global distribution of the Pn cluster, we used metagenomic fragment recruitment (>90% identity) from the *Tara* metagenomic data sets (see Experimental procedures section). Figure 4B shows that this cluster was only found in the Mediterranean Sea and the North Atlantic Ocean with higher prevalence in surface waters. Furthermore, even within the Mediterranean Sea, results showed a higher presence of this gene cluster in the Eastern basin rather than in the Western Mediterranean (Fig. 4C). These results fit with the Mediterranean Sea oligotrophy and limiting P concentrations compared with the global ocean and with the ultraoligotrophic nature of the Eastern basin (Tanhua *et al*., 2013), and suggests a role of P availability in the divergence and different adaptation of these genomospecies to a specific niche as seems to be the case of picocyanobacteria (Coleman and Chisholm, 2010).

On the other hand, gWID had genes involved in oligosaccharides and oligopeptide transport systems (Tam and Saier, 1993) that were more abundant than in gMED. Another relevant difference was the presence of the carbon monoxide dehydrogenase, which catalyses the oxidation of CO to CO_2_ as an energy source, a mechanism already described in some SAR11 isolates (Grote *et al*., 2012) (Fig. 4A). Another difference that we found in a genomic island (not found in gMED genomes) was a cluster of genes involved in aerobic purine degradation (Fig. S10A). Nucleic acids are an important component of the dissolved organic nitrogen pool (Berman and Bronk, 2003). The ability to utilize purines as nitrogen and carbon sources is a metabolic capability that could contribute to their success in different marine ecosystems. To evaluate the global distribution of this island, we used again metagenomic fragment recruitment (>90% identity) from the different *Tara* oceans provinces (Fig. S10B). The data suggest a correlation between the genomic island distribution and nutrient concentration. In nutrient‐rich areas such as the Southern Ocean (Pollard *et al*., 2006), the island is not present while it recruited the most in the Red Sea, notorious for its ultraoligotrophic nature (Kürten *et al*., 2019). In the Mediterranean Sea, we also observed lower recruitment values, probably because there the limiting factor is P (Fig. S10B).

### *Sequence microdiversity of gMED and gWID*

After analysing the differences in the genomic content, we investigated the sequence divergence of both genomospecies (gMED and gWID). Specifically, we determined the ratio of synonymous to nonsynonymous single‐nucleotide polymorphisms (SNPs) for the three most complete genomes of each genomospecies (pN/pS) across different metagenomic samples. We observed similar median values within each genomospecies along all the metagenomes. Furthermore, genomic comparisons between both groups allowed us to differentiate between shared (core) and differential (flexible) gene content. The gMED representative AG‐414‐O11 core genome pN/pS value was 0.27 ± 0.04 and 0.35 ± 0.05 for the flexible, while gWID HIMB083 had lower diversity values (0.16 ± 0.02 and 0.14 ± 0.03 for the core and flexible genome respectively). We detected a high rate of synonymous SNPs which may indicate that strong purifying selection is acting on both genomospecies. The pN/pS values for gMED are nearly double those of gWID, which seems to have less population genomic diversity.

Although only a few genes appeared under positive selection (pN/pS > 1), we obtained information about all such genes in each sample. For gMED, genes or orthologous groups with the highest pN/pS ratios were related to ‘Cell Wall Components’, ‘Carotenoids’, proteorhodopsin, and ‘Global Redox‐Responding two‐component system’ (RegB/RegA), involved in numerous energy‐generating and energy‐utilizing processes (Wu and Bauer, 2008). In contrast, for the less‐diversified gWID, genes related to functional classification ‘Amino Acids and Derivatives’ as well as ‘Respiration’ were the ones under positive selection.

### *Horizontal versus vertical microdiversity*

**Figure 5:**
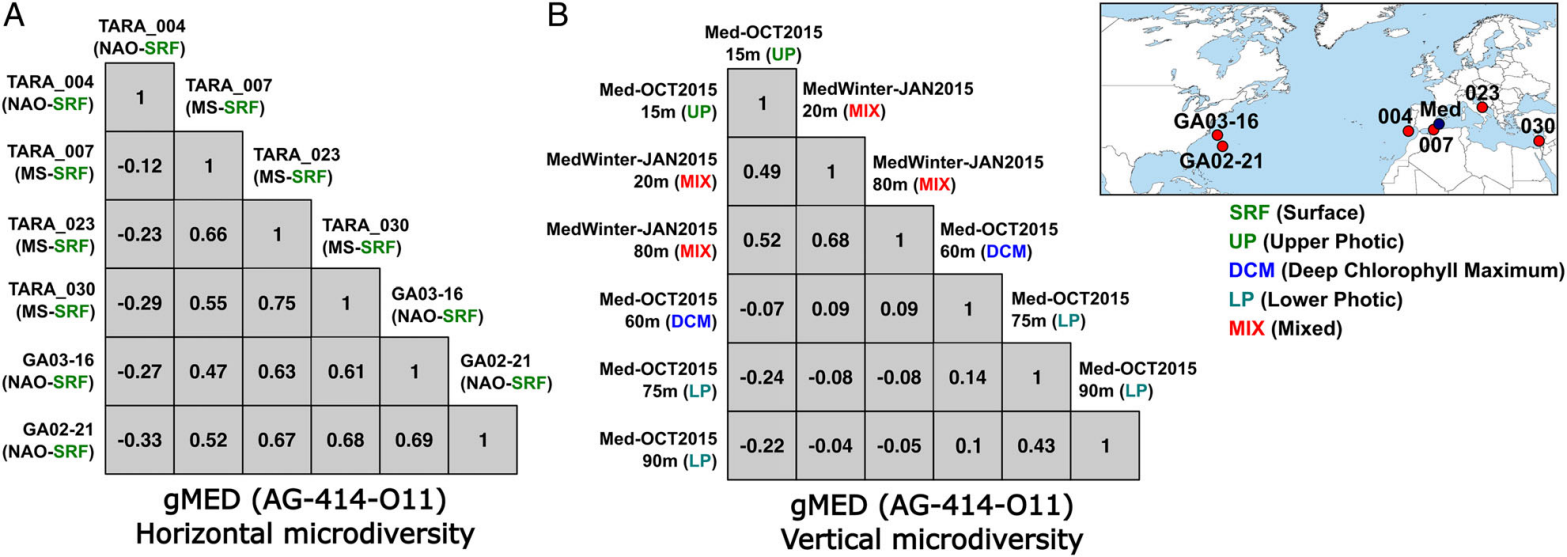
Correlograms of SNPs position conservation along the reference genome of gMED (AG‐414‐O11) across the different metagenomes (microdiversity). Linear Pearson's correlation coefficients range from −1 to 1, taking 1 as the reference value of a metagenome against itself. (A) Horizontal and (B) vertical microdiversity through the water column in a single location. Map shows the location of the samples used in the analyses.

It is obvious from the recruitment plots that the Pelagibacterales possess a large sequence diversity at the population level (Figs 3C and S8B). We wanted to assess the diversity that can be found for gMED and gWID when they were compared with individual reads from metagenomes at ≥1% divergence, that is, within the same clonal frame (Milkman and Bridges, 1990). Illumina sequencing errors were curated by filtering only polymorphisms present >4 times. The analyses were performed with the three most complete genomes of each genomospecies, but only one is shown since similar patterns were observed in all of them. We normalized the conservation of the SNPs along the reference genome across the different metagenomes as a range from −1 to 1, taking 1 as the reference value of a metagenome against itself. A remarkable population diversity conservation was found among all the samples despite the geographical distance, suggesting little influence of biogeography at this level of microdiversity (Fig. 5A). Despite the global distribution of gWID, its genomic diversity was not higher (Fig. S10A). In fact, the genomic variation that we found in samples throughout the global ocean was similar or even lower than we could find for gMED across the Mediterranean (Fig. S11A). Interestingly, for gMED, depth‐dependent genomic variation at a single location showed more dramatic changes than geographic (horizontal) variation (Fig. 5B). We found three genomic variants through the water column that corresponded to the three layers previously described (Haro‐Moreno *et al*., 2018) [15 m or upper photic (UP); 60 m or DCM; and 90 m or lower photic]. In addition, the variant from the UP was the most similar to the variant found in the mixed water column at both depths (Fig. 5B). In the same way, gWID, in spite of its global distribution, had as much diversity across the water column of the Red Sea (Haroon *et al*., 2016) as across the geographic span of the *Tara* samples (Fig. S11B).

## Conclusions

The genomic diversity within the SAR11 clade, be it at the levels of genera, species or clonal frames, is astounding. Illustrating the complexity of interactions and subtle micro‐adaptations that are required to exploit even the relatively homogeneous offshore oligotrophic marine waters. Overall, this pattern seems to be an extreme example of the ‘Paradox of the plankton’ (Kenitz *et al*., 2013) that postulates a contradiction between high biodiversity of planktonic organisms and the low variety of resources in aquatic ecosystems in view of the competitive exclusion principle.

Our results also illustrate a remarkable example of the uneven representation of different but closely related microbes. While a few are massively abundant, others are present in small numbers but still play a significant role in ecosystem functioning depending on environmental variations. This normal distribution curve of the abundance of different species is actually a widespread feature of ecosystems and appears to be applicable to the Pelagibacterales as well. Our data also support the concept of the ‘rare biosphere’ (Sogin *et al*., 2006), that is, microbes that are present in small amounts but still play a significant role in the ecosystem functioning given the proper conditions. Furthermore, population diversity conservation, despite the geographical distance together with a strong purifying selection of the core genome, suggests that the differential gene content among genomospecies was the main driver behind their distribution.

Although this study is a step further in understanding the population structure of one of the most ecologically dominant groups on the planet combining genomic, metagenomics and data from environmental distribution, further work will be needed to obtain more cultured isolates around the world and understand the molecular basis that underlies their evolution and distribution.

## Experimental procedures

### *Sample collection and processing*

For SCGs, a surface seawater sample was collected on April 4, 2017 from the Blanes Bay Microbial Observatory, located in the North‐Western Mediterranean Sea (41°40′13.5″ N 2°48′00.6″ E; 2.7 miles offshore). The sample was kept at 4°C and immediately processed for single‐cell sorting. Prior to sorting, all the reagents and materials were decontaminated following a strict procedure described in Rinke and colleagues (2014), although a few modifications were made (Martinez‐Hernandez *et al*., 2017, 2018). A BD Influx sorter (Bector Dickinson, San Jose, CA) was used, calibrated as described in Martinez‐Hernandez and colleagues (2017), using gates corresponding to the bacterial fraction (Swan *et al*., 2011; Rinke *et al*., 2014). Cells from 1 ml of fresh, natural sample were stained with SYBR‐Gold (Invitrogen) at a final concentration of 0.5×. Then, the sample was incubated for 15 min at room temperature.

A total of 1992 single cells were sorted on 394‐well plates. Multiple‐displacement amplification (MDA) was done using phi29 DNA polymerase (ref. M0269L; New England Biolab) in six plates. In another single plate, the new thermostable phi29 DNA polymerase (Stepanauskas *et al*., 2017) was used. For this new enzyme, the final concentration of whole‐genome amplification reactions was as follows: 0.2 U μl^–1^ Equiphi29 polymerase (Thermo Fisher Scientific, ref. A39391), 1× Equiphi29 reaction buffer, 0.4 mM each dNTP (New England BioLabs), 10 mM dithiothreitol, 40 μM random heptamers with two 3′‐terminal phosphorothioated nucleotide bonds (Integrated DNA Technologies) and 1 μM SYTO‐9 (Thermo Fisher Scientific). These reactions were performed at 45°C for 3–4 h in a fluorimeter (CLARIOstar, BMG) and inactivated by incubation at 75°C for 15 min.

Positive MDA products with both enzymes were diluted to a final concentration of 0.1× in TE buffer (10 mM Tris, 1 mM EDTA; pH 8.0) and transferred to a new 96‐well plate. Only 300 SAGs were screened by polymerase chain reaction targeting the 16S rRNA gene with primers 341F and 907R (Schäfer *et al*., 2000) with cycling conditions as described (Martínez‐García *et al*., 2007). 16S rRNA sequences were taxonomically classified using the RDP (Cole *et al*., 2014) and SILVA (Quast *et al*., 2013) databases. In the end, only 87 SAGs affiliated to the SAR11 clade.

Fifty SAR11‐positive SAGs were randomly selected and sequenced using an Illumina Hiseq‐4000 (150 bp, paired‐end read) (Novogene, Hong‐Kong) following construction of a Nextera XT library as per the manufacturer's protocol. For each SAG, approximately 1 Gb was sequenced. Reads were trimmed and assembled using Trimmomatic v0.36 (Bolger *et al*., 2014) and SPAdes v3.11.1 (Bankevich *et al*., 2012), with the single‐cell option respectively. Resulting genes on the assembled contigs were predicted using Prodigal v2.6 (Hyatt *et al*., 2010). tRNA and rRNA genes were predicted using tRNAscan‐SE v1.4 (Lowe and Eddy, 1996), ssu‐align v0.1.1 (Nawrocki, 2009) and meta‐rna (Huang *et al*., 2009). Predicted protein sequences were compared against the NCBI nr database using DIAMOND (Buchfink *et al*., 2015), and against COG (Tatusov *et al*., 2001) and TIGFRAM (Haft *et al*., 2001) using HMMscan v3.1b2 (Eddy, 2011) for taxonomic and functional annotation. Manual curation was performed in order to remove small and overlapping scaffolds.

### *Phylogenomic classification of the SAR11 clade*

All the available genomes belonging to the different SAR11 clades were downloaded from the NCBI database (accessed in September 2018). Additionally, SAGs obtained in (Berube *et al*., 2018) were also included in the analysis. To guarantee a robust phylogenomic tree, we used CheckM (Parks *et al*., 2015) to remove low‐quality sequences, and only those genomes with a completeness > 50% and contamination < 5% were kept. Only 47 out of 50 SAGs collected in this study were above our quality criteria. Using Phylophlan (Segata *et al*., 2013), a total of 232 genes were used to classify the sequences phylogenomically. Three genomes of *Rickettsia* spp. were used as an outgroup. The resulting tree was analysed using iTOL (Letunic and Bork, 2016). Subclades were defined starting from the phylogenomic tree topology. We used the median distance between nodes and cophenetic correlation coefficient (interval comprised between 0 and 2) in order to define them, similarly to the approach used by Ragonnet‐Cronin and colleagues (2013). The well‐established SAR11 nomenclature within subclades and phylotypes was followed.

### *ITS phylogeny of the SAR11 clade*

Phylotype classification based on the ITS was inferred using the neighbour‐joining approach in MEGA7 (Kumar *et al*., 2016), with 1000 bootstraps and the Jukes–Cantor model of substitution. Phylotype assignment followed existing ITS nomenclature (García‐Martínez and Rodríguez‐Valera, 2000; Brown *et al*., 2012; Ngugi and Stingl, 2012; Jimenez‐Infante *et al*., 2017).

### *Genomic pairwise comparison*

ANI and AAI between a pair of genomes were calculated using the JSpecies with default parameters (Richter and Rossello‐Mora, 2009) and CompareM (https://github.com/dparks1134/CompareM) software packages respectively.

### *Pangenome analysis*

The pangenome of each genomospecies (gMED and gWID) was calculated by clustering all the predicted proteomes of each genome belonging to a specific genomospecies with a 90% amino acid identity cut‐off using CD‐HIT (Huang *et al*., 2010). Paralogs were removed from all clusters; next, CD‐HIT‐2D (Huang *et al*., 2010) was used to compare both pangenomes removing those clusters that were present in both pangenomes (cut‐off of 75% identity) and leaving for further analysis those sequences that were unique. Finally, those proteins were functionally annotated against the SEED Subsystems database (Overbeek *et al*., 2005), using DIAMOND (blastp option, top hit, ≥ 50% identity, ≥ 50% alignment length, *E*‐value <10^−5^).

### *Metagenomic fragment recruitment and SAR11 biogeography*

Metagenomes from different environmental datasets were used to study SAR11 distribution. Raw reads from *Tara* Oceans expedition (Sunagawa *et al*., 2015) were downloaded from the European Nucleotide Archive (PRJEB1787). Raw reads from GEOTRACES and HOT/BATS (Biller *et al*., 2018) expeditions were downloaded from NCBI‐SRA (PRJNA385854 and PRJNA385855). A metagenomic data set from a Mediterranean Sea profile (Haro‐Moreno *et al*., 2018) was also downloaded from NCBI‐SRA (BioProject accession number PRJNA352798). Prior to recruitment, the complete ribosomal operon gene cluster was manually removed from each SAR11 genome sequence. Metagenomic reads were trimmed using Trimmomatic v0.36 (Bolger *et al*., 2014) and only those reads with a phred score ≥ 30, ≥ 50 bp long and with no ambiguous bases (Ns) were kept. These high‐quality metagenomic reads were then aligned using BLASTN (Altschul *et al*., 1997), using a cut‐off of 98% nucleotide identity over a minimum alignment length of 50 nucleotides. We required ≥ 70% of each genome to be covered by reads. They were used to compute the RPKG (reads recruited per Kb of genome per Gb of metagenome) values that provide a normalized number comparable across various metagenomes. Since different data sets with different read lengths (Illumina Hiseq 2 × 100 and 2 × 150 bp) were used for the recruitment, each metagenome was also normalized, dividing the size of the database by its average read size. Genomes that recruited less than three RPKG were considered not present in the sample. The resulting RPKG values were used to cluster the SAR11 genomes that recruited similarly (average linkage, Euclidean distance), and delineate different geographic variants (genomospecies) within subclades. The same parameter (RPKG) was used for the global distribution of the Pn cluster with an identity > 90% from the Tara metagenomic data set.

To investigate patterns of co‐occurrence, Pearson's *r* linear correlations of absolute RPKG abundances were computed among genomospecies using the ggcorrplot package (https://github.com/kassambara/ggcorrplot) in R. Only correlations with a significance level (*p*‐value) less than 0.05 were considered. Additionally, correlations between physicochemical parameters [${\mathrm{PO}}_{4}^{3-}$, ${\mathrm{NO}}_{2}^{-}$, ${\mathrm{NO}}_{3}^{-}$, dissolved inorganic carbon, O_2_, salinity (Sal), temperature (T), depth and latitude (Lat)] and genomospecies abundances across GEOTRACES GA02 cruise samples were achieved conducting a non‐metric multidimensional scaling (NMDS) analysis with the vegan package (Dixon, 2003) in R. Significant correlation of environmental parameters to sample ordination was tested with the envfit function in vegan (1000 permutations), and only physicochemical parameters with a *p*‐value <0.05 were included.

### *Linear metagenomic fragment recruitments*

The same high‐quality metagenomic reads described above were used to graphically represent their mapping along a reference genome. Reads were aligned using BLASTN (Altschul *et al*., 1997), using a cut‐off of 70% nucleotide identity over a minimum alignment length of 50 nucleotides. The resulting alignments, together with the distribution of the reads according to the identity of the alignment (histogram) were plotted using the ggplot2 package in R.

### *Microdiversity*

High‐quality trimmed metagenomic reads were aligned against SAR11 reference genomes using Bowtie2 using sensitive‐local mode (Langmead and Salzberg, 2012). Results were converted in mpileup format using samtools (Li *et al*., 2009), and used to carry out the SNP predictions. Analyses focused into nucleotide substitutions, while point insertions (indels) and deletions were not considered. In order to provide enough consistency in the analysis two thresholds were hence applied: (i) the read coverage at each position was set above 30×; (ii) further variants that were eventually identified must have recurred at least in 20% of all bases called at each position. Only SNPs that passed this filter were considered to estimate the ratio of synonymous and nonsynonymous SNPs within a genome (pN/pS).

## Author contributions

MLP conceived the study. FMH, JRG, MLG and OF collected and processed the sample. JHM, FRV, RS and MLP analysed the data. JHM, FRV, MMG and MLP contributed to write the manuscript.

## Conflict of interests.

The authors declare that they have no competing interests.

## Supporting information


**Fig. S1:** Neighbour‐joining phylogenetic tree (145 sequences, 1000 bootstraps, Jukes–Cantor distance correction) of the internal space transcriber (ITS) located between the 16S and the 23S rRNA operon. Nomenclature for phylotype assignments is derived from Brown and colleagues (2012), Ngugi and Stingl (2012) and Jimenez‐Infante and colleagues (2017). Reference genomes, reference ITS sequences and genomes analysed in this study are shown in blue, green and red, respectively.
**Fig. S2:** Pairwise comparison among the SAR11 genomes using both amino acid identity (AAI) and average nucleotide identity (ANI). Rectangles with continuous and dotted line delimit subclades and genomospecies, respectively.
**Fig. S3:** Pairwise comparison among SAGs and isolated reference genomes of the SAR11 IV and V subclades. Genomes of the Pelagibacterales order (clades Ia to IIIa) were included in the analysis and are highlighted with a green rectangle. A) Average nucleotide identity (ANI) distance matrix. B) Percentage of the genome aligned (coverage) during the ANI analysis. C) Average amino acid identity (AAI) distance matrix. D) Percentage of proteins shared during the AAI analysis.
**Fig. S4:** Comparison of the abundance of 185 SAR11 genomes (we excluded the clades IV and V, whose affiliation to the SAR11 clade is controversial) in 20 randomly selected TARA metagenomes. RPKG values obtained after the removal of the ribosomal RNA operon (x axis) were compared to those obtained recruiting the whole genome (y axis). Only reads recruiting >98% identity with an alignment >50 bp long were considered. Dashed red lines represent the threshold of 3 RPKG applied to discriminate between presence (>3) or absence (<3) of a genome in a sample. Dashed blue lines indicate the ratio between RPKG values. The area framed in orange includes all those genomes which, if the ribosomal operon had not been eliminated, would have given a false positive (*ca*. 32.5%).
**Fig. S5:** Clustering of the SAR11 genomes recruited along several GEOTRACES metagenomic samples, based on their abundance values (in RPKG). A representation of the GEOTRACES cruises is shown at the bottom of the figure. The different genomospecies obtained after clustering are indicated at the bottom of each heatmap.
**Fig. S6:** Clustering of the SAR11 genomes recruited along several *Tara* metagenomic samples, based on their abundance values (in RPKG). The different genomospecies obtained after clustering are indicated at the top of the heatmap. Coloured rectangles on the right indicate the oceanic region from which those samples were collected. A representation of the *Tara* cruises is shown at the bottom of the figure.
**Fig. S7:** Bar‐plots showing the recruitment values in RPKGs at 98% nucleotide identity (x‐axis), of only those genomospecies that recruited >3 RPKG in at least one of the Mediterranean depth profile samples (y‐axis), collected during summer (stratified water column) and winter (mixed water column). Red dotted line indicates the threshold (3 RPKG) used to discriminate between presence (>3) or absence (<3) in the sample.
**Fig. S8: A**, Metagenomic recruitment of genomospecies Ia.3/VII (gMED), Ia.3/VIII, Ia.4/II, Ib.2/I and Ib.1/III in the Bermuda Atlantic Time‐series Study (BATS) during two consecutive years. **B**, Recruitment plot of the SAR11 genome AG‐430‐E20 (Ia.3/VII gMED) at three different dates during the BATS time‐series: August 2003 (1); January 2004 (2) and August 2004 (3). Numbers between parenthesis indicate RPKG values at 98% identity. Histogram on the right shows the relative percentage of aligned reads in intervals of 1% identity. Black dashed line indicates the species threshold (95%). **C**, Similar to A., but using the metagenomes collected during two consecutive years in the Hawai'i Ocean Time‐series (HOT). Only genomospecies that recruited more than 3 RPKGs at 98% identity are shown.
**Fig. S9:** tBlastX genome comparison of three representatives of genomospecies Ia.3/VII (gMED), Ia.3/V (gWID) and Ia.3/I. Only the Genomic Island containing the phosphate (blue genes) and phosphonate acquisition (yellow), regulation (green) and degradation (cyan) is shown.
**Fig. S10:** A) An overview of the characteristic metabolism encoded in the flexible genome of members of Ia.3/V (gWID), compared to Ia.3/VII (gMED). Genes highlighted in blue are located together in a single operon (denoted as purine degradation cluster). B) Boxplot indicating the recruitment values (y‐axis) of the purine degradation cluster at three depths (SRF – Surface, DCM – Depth Chlorophyll Maximum, MES ‐ Mesopelagic) among *Tara* regions.
**Fig. S11:** Correlograms of single nucleotide polymorphisms (SNPs) position conservation along the reference genome of HIMB083 (Ia.3/V, gWID) HIMB083 across the different metagenomes (Microdiversity). Linear Pearson's correlation coefficients range from ‐1 to 1, taking 1 as the reference value of a metagenome against itself. **A**, Horizontal and **B**, vertical microdiversity through the water column in a single location. Map shows the location of the samples used in the analyses.

**Table S1** List of SAR11 single‐amplified genomes (SAGs) collected in this study, together with some genomic properties.

**Table S2** List of all SAR11 sequences used in this work. The table shows the genome completeness (%), degree of contamination (%) and strain heterogeneity (%), computed using CheckM. Highlighted in grey are those genomes excluded for further analyses

**Table S3** Average nucleotide identity (ANI) and Average amino acid identity (AAI) within SAR11 clades, subclades and genomospecies

**Table S4** Recruitment values, expressed in RPKGs, of the SAR11 genomes. Recruited genomes were previously modified removing the 16S, 5S and 23S rRNA operon. For each metagenome, the accession number, depth and date of the collection is provided. Genomes are classified according to the subclade and genomospecies descriptions included in Fig. 1. A and B) Recruitment values for the Hawaii Ocean Timeseries (HOT) and the Bermuda Ocean Timeseries (BATS) (NCBI BioProject PRJNA385855). C) Recruitment values for the MEDIMAX expedition (NCBI BioProject PRJNA352798). D) Recruitment values for the Tara Oceans expedition (ENA BioProject PRJEB1787). E) Recruitment values for the GEOTRACES expedition (NCBI BioProject PRJNA385854).

## Data Availability

Single‐cell genomes have been deposited under BioProject PRJNA473343.
