# Supplementary material for: Ecogenomics of the SAR11 clade
Source: Environ Microbiol. 2019 Dec 25;22(5):1748–63. doi: 10.1111/1462-2920.14896 (PMC7318151; doi:10.1111/1462-2920.14896)

Ecogenomics of the SAR11 clade

Jose M. Haro-Moreno[1], Francisco Rodriguez-Valera[1,2], Riccardo Rosselli[1,3], Francisco Martinez-Hernandez[4], Juan J. Roda-Garcia[1], Monica Lluesma Gomez[4], Oscar Fornas[5], Manuel Martinez-Garcia[4] and Mario López-Pérez[1,*]

[1]Evolutionary Genomics Group, División de Microbiología, Universidad Miguel Hernández, Apartado 18, San Juan 03550, Alicante, Spain. [2]Laboratory for Theoretical and Computer Research on Biological Macromolecules and Genomes, Moscow Institute of Physics and Technology, Moscow, Russia. [3]Department of Marine Microbiology and Biogeochemistry, Royal Netherlands Institute for Sea Research (NIOZ), The Netherlands. [4]Department of Physiology, Genetics, and Microbiology, University of Alicante, Alicante, Spain. [5]Flow Cytometry Unit: Pompeu Fabra University (UPF) and Centre for Genomic Regulation (CRG), The Barcelona Institute for Sciences and Technology (BIST), Barcelona, Spain.

[*]Corresponding author: mario.lopezp@umh.es

SUPPLEMENTARY INFORMATION

Fig S1: Neighbour-joining phylogenetic tree (145 sequences, 1000 bootstraps, Jukes-Cantor distance correction) of the internal space transcriber (ITS) located between the 16S and the 23S rRNA operon. Nomenclature for phylotype assignments is derived from Brown et al. (2012), Ngugi and Stingl (2012) and Jimenez-Infante (2017). Reference genomes, reference ITS sequences and genomes analysed in this study are shown in blue, green and red, respectively.

Fig. S2: Pairwise comparison among the SAR11 genomes using both amino acid identity (AAI) and average nucleotide identity (ANI) distances. Rectangles with continuous and dotted line delimit subclades and genomospecies, respectively.

Fig S3: Pairwise comparison among SAGs and isolated reference genomes of the SAR11 IV and V subclades. Genomes of the Pelagibacterales order (clades Ia to IIIa) were included in the analysis and are highlighted with a green rectangle. A) Average nucleotide identity (ANI) distance matrix. B) Percentage of the genome aligned (coverage) during the ANI analysis. C) Average amino acid identity (AAI) distance matrix. D) Percentage of proteins shared during the AAI analysis.

Fig. S4: Comparison of the abundance of 185 SAR11 genomes (we excluded the clades IV and V, whose affiliation to the SAR11 clade is controversial) in 20 randomly selected TARA metagenomes. RPKG values obtained after the removal of the ribosomal RNA operon (x axis) were compared to those obtained recruiting the whole genome (y axis). Only reads recruiting >98% identity with an alignment >50 bp long were considered. Dashed red lines represent the threshold of 3 RPKG applied to discriminate between presence (>3) or absence (<3) of a genome in a sample. Dashed blue lines indicate the ratio between RPKG values. The area framed in orange includes all those genomes which, if the ribosomal operon had not been eliminated, would have given a false positive (*ca.* 32.5%).

Fig. S5: Clustering of the SAR11 genomes recruited along several GEOTRACES metagenomic samples, based on their abundance values (in RPKG). A representation of the GEOTRACES cruises is shown at the bottom of the figure. The different genomospecies obtained after clustering are indicated at the bottom of each heatmap.

Fig. S6: Clustering of the SAR11 genomes recruited along several *Tara* metagenomic samples, based on their abundance values (in RPKG). The different genomospecies obtained after clustering are indicated at the top of the heatmap. Coloured rectangles on the right indicate the oceanic region from which those samples were collected. A representation of the *Tara* cruises is shown at the bottom of the figure.

Fig. S7: Bar-plots showing the recruitment values in RPKGs at 98% nucleotide identity (x-axis), of only those genomospecies that recruited >3 RPKG in at least one of the Mediterranean depth profile samples (y-axis), collected during summer (stratified water column) and winter (mixed water column). Red dotted line indicates the threshold (3 RPKG) used to discriminate between presence (>3) or absence (<3) in the sample.

Fig. S8: **A**, Metagenomic recruitment of genomospecies Ia.3/VII (gMED), Ia.3/VIII, Ia.4/II, Ib.2/I and Ib.1/III in the Bermuda Atlantic Time-series Study (BATS) during two consecutive years. **B**, Recruitment plot of the SAR11 genome AG-430-E20 (Ia.3/VII gMED) at three different dates during the BATS time-series: August 2003 (1); January 2004 (2) and August 2004 (3). Numbers between parenthesis indicate RPKG values at 98% identity. Histogram on the right shows the relative percentage of aligned reads in intervals of 1% identity. Black dashed line indicates the species threshold (95%). **C**, Similar to A., but using the metagenomes collected during two consecutive years in the Hawai'i Ocean Time-series (HOT). Only genomospecies that recruited more than 3 RPKGs at 98% identity are shown.

Fig. S9: tBlastX genome comparison of three representatives of genomospecies Ia.3/VII (gMED), Ia.3/V (gWID) and Ia.3/I. Only the Genomic Island containing the phosphate

(blue genes) and phosphonate acquisition (yellow), regulation (green) and degradation (cyan) is shown.

Fig S10: A) An overview of the characteristic metabolism encoded in the flexible genome of members of Ia.3/V (gWID), compared to Ia.3/VII (gMED). Genes highlighted in blue are located together in a single operon (denoted as purine degradation cluster). B) Boxplot indicating the recruitment values (x-axis) of the purine degradation cluster at three depths (SRF – Surface, DCM – Depth Chlorophyll Maximum, MES - Mesopelagic) among *Tara* regions.

Fig. S11: Correlograms of single nucleotide polymorphisms (SNPs) position conservation along the reference genome of HIMB083 (Ia.3/V, gWID) HIMB083 across the different metagenomes (Microdiversity). Linear Pearson's correlation coefficients range from -1 to 1, taking 1 as the reference value of a metagenome against itself. **A**, Horizontal and **B**, vertical microdiversity through the water column in a single location. Map shows the location of the samples used in the analyses.

Table S1: List of SAR11 single-amplified genomes (SAGs) sequenced for this study, together with some genomic properties and their clade, subclade and genomospecies affiliation.

Table S2: List of all SAR11 sequences used in this work. The table shows the genome completeness (%), degree of contamination (%) and strain heterogeneity (%), computed using CheckM. Highlighted in grey are those genomes excluded for further analyses.

Table S3: Average nucleotide identity (ANI) and coverage; and Average amino acid identity (AAI) within SAR11 clades, subclades and genomospecies.

Table S4: Recruitment values, expressed in RPKGs, of the SAR11 genomes. Recruited genomes were previously modified removing the 16S, 5S and 23S rRNA operon. For

each metagenome, the accession number, depth and date of the collection is provided. Genomes are classified according to the subclade and genomospecies descriptions included in Fig 1. A and B) Recruitment values for the Hawaii Ocean Timeseries (HOT) and the Bermuda Ocean Timeseries (BATS) (NCBI BioProject PRJNA385855). C) Recruitment values for the MEDIMAX expedition (NCBI BioProject PRJNA352798). D) Recruitment values for the *Tara Oceans* expedition (ENA BioProject PRJEB1787). E) Recruitment values for the GEOTRACES expedition (NCBI BioProject PRJNA385854).

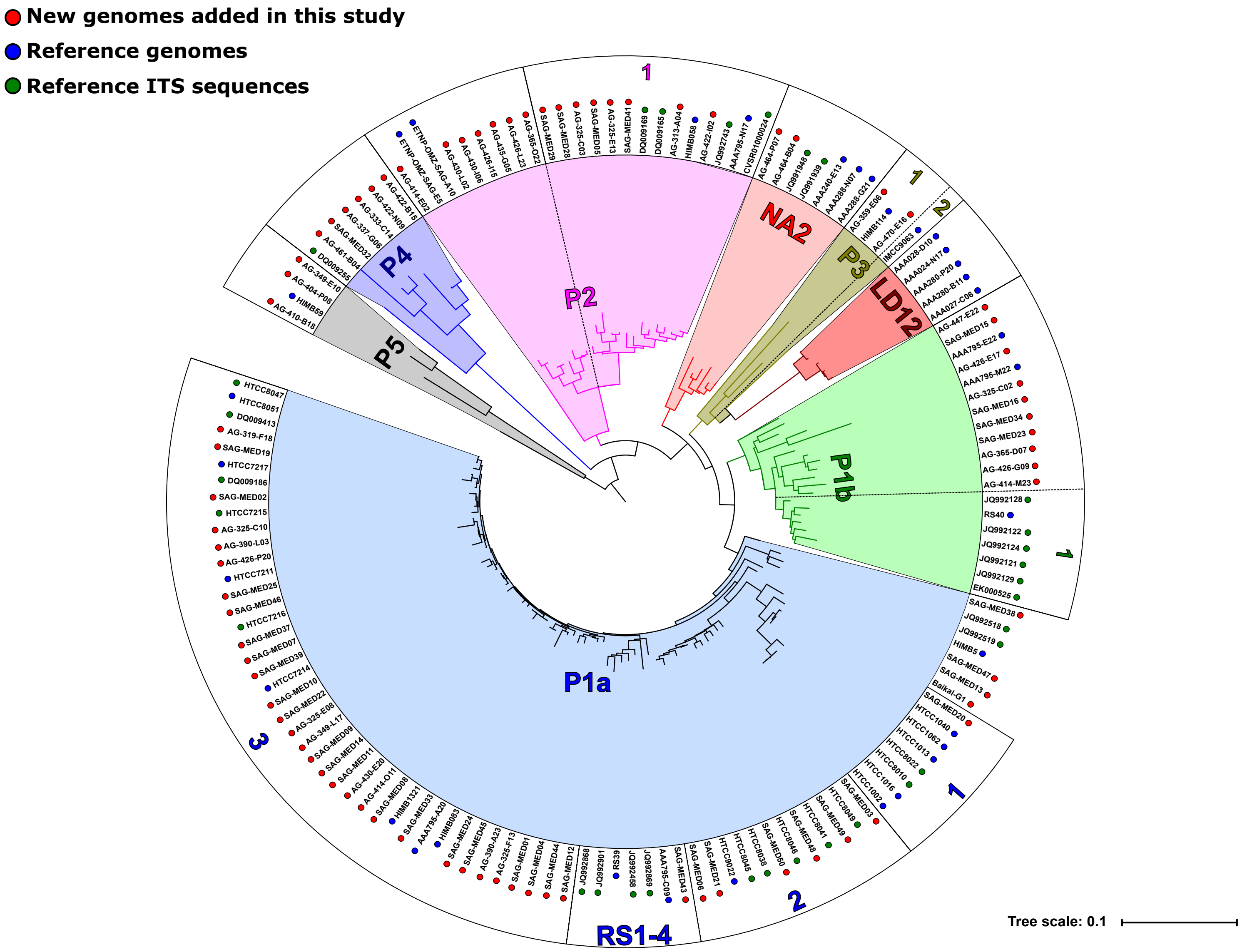
New genomes added in this study
Reference genomes
Reference ITS sequences
P1a
P1b
P2
P3
P4
P5
NA2
LD12
RS1-4
1
2
3
Tree scale: 0.1

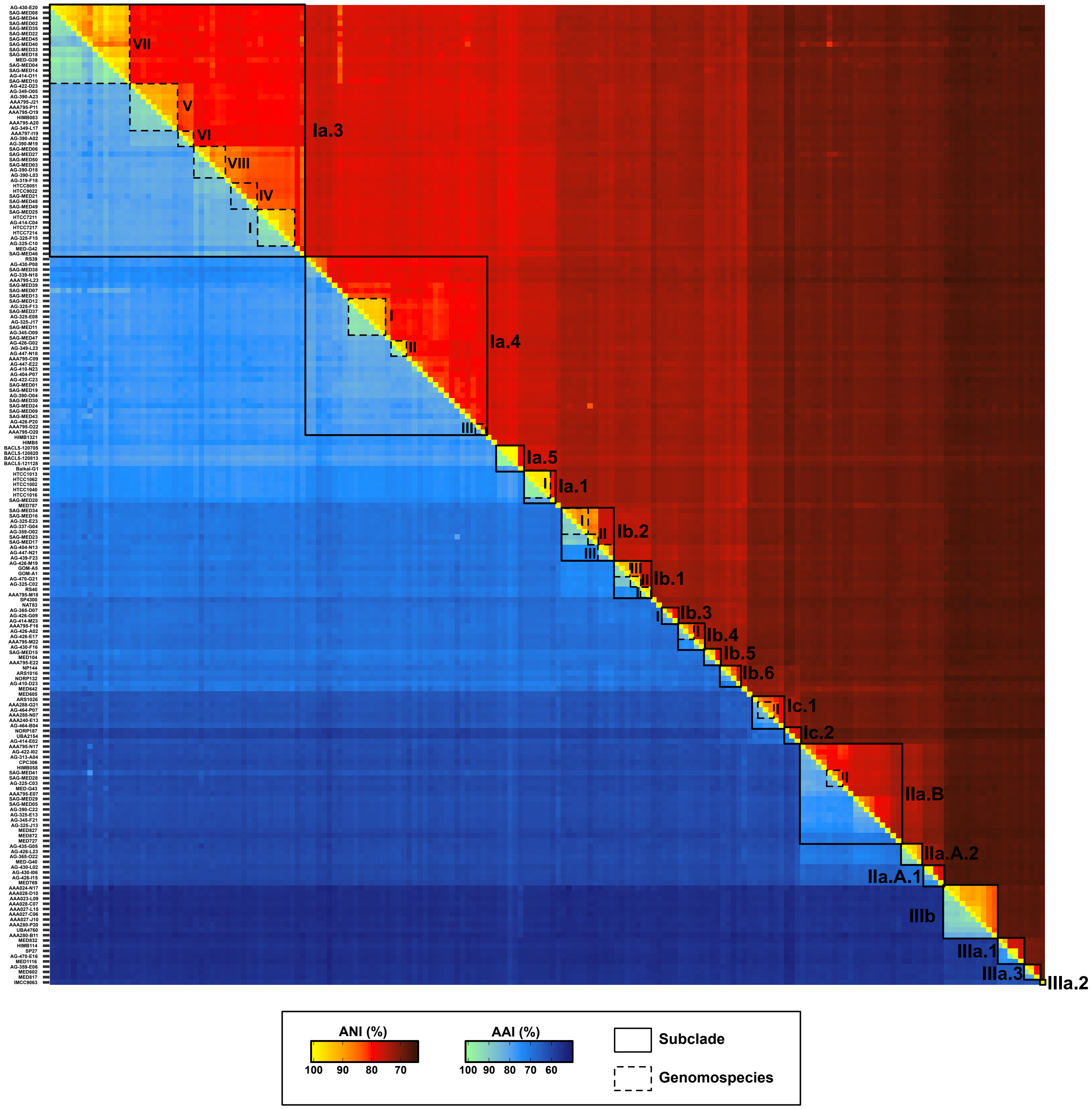

Ia.3
VII
V
VI
VIII
IV
I
Ia.4
I
II
III
Ia.5
Ia.1
I
Ib.2
I
II
III
Ib.1
III
II
Ib.3
I
Ib.4
I
Ib.5
Ib.6
Ic.1
II
Ic.2
IIa.B
II
IIa.A.2
IIa.A.1
IIIb
IIIa.1
IIIa.3
IIIa.2
ANI (%)
100 90 80 70
AAI (%)
100 90 80 70 60
Subclade
Genomospecies

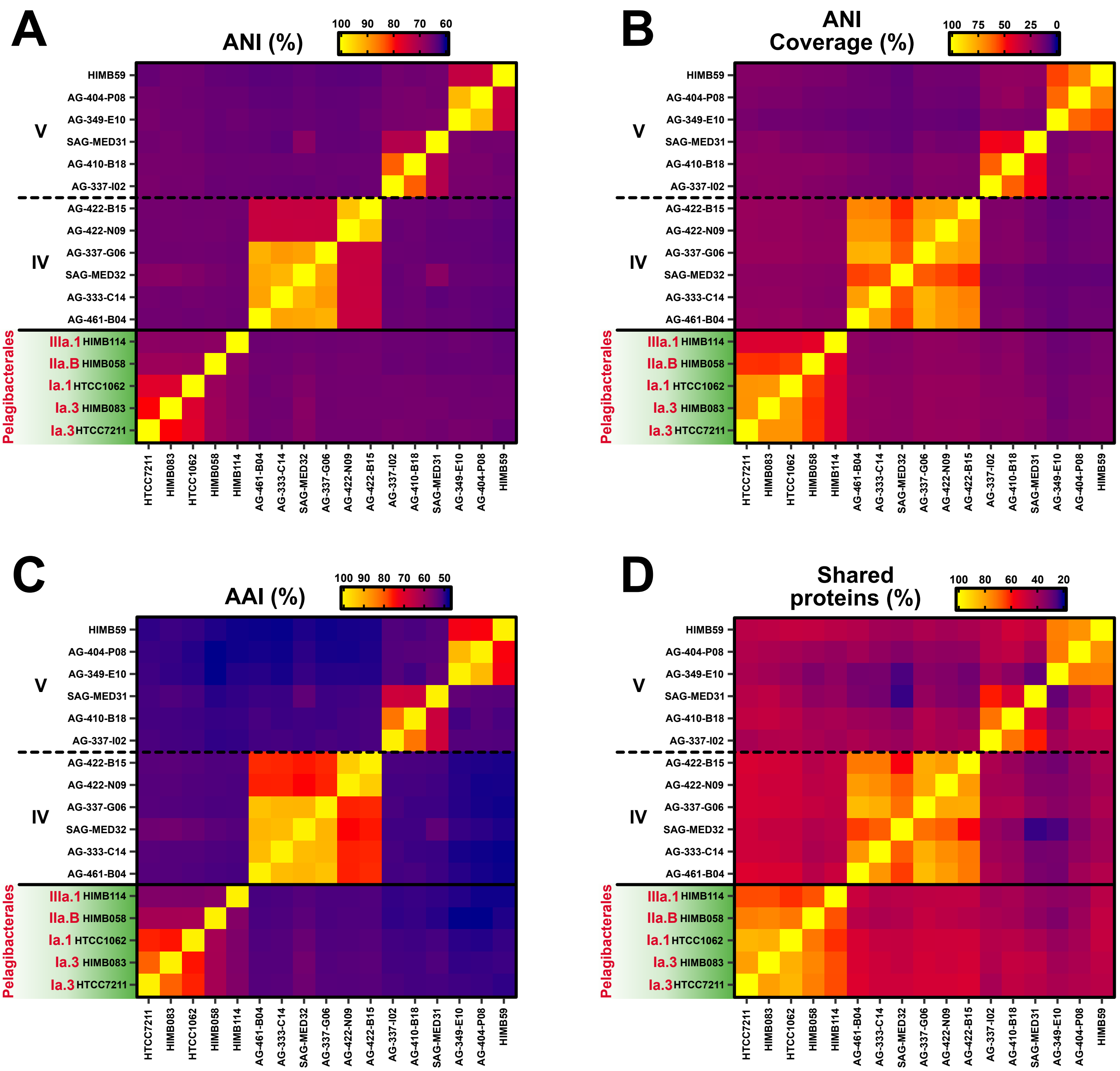
A
ANI (%)
100 90 80 70 60
HIMB59
AG-404-P08
AG-349-E10
SAG-MED31
AG-410-B18
AG-337-I02
AG-422-B15
AG-422-N09
AG-337-G06
SAG-MED32
AG-333-C14
AG-461-B04
IIIa.1 HIMB114
IIa.B HIMB058
Ia.1 HTCC1062
Ia.3 HIMB083
Ia.3 HTCC7211
V
IV
Pelagibacterales
HTCC7211
HIMB083
HTCC1062
HIMB058
HIMB114
AG-461-B04
AG-333-C14
SAG-MED32
AG-337-G06
AG-422-N09
AG-422-B15
AG-337-I02
AG-410-B18
SAG-MED31
AG-349-E10
AG-404-P08
HIMB59
B
ANI
Coverage (%)
100 75 50 25 0
C
AAI (%)
100 90 80 70 60 50
D
Shared
proteins (%)
100 80 60 40 20

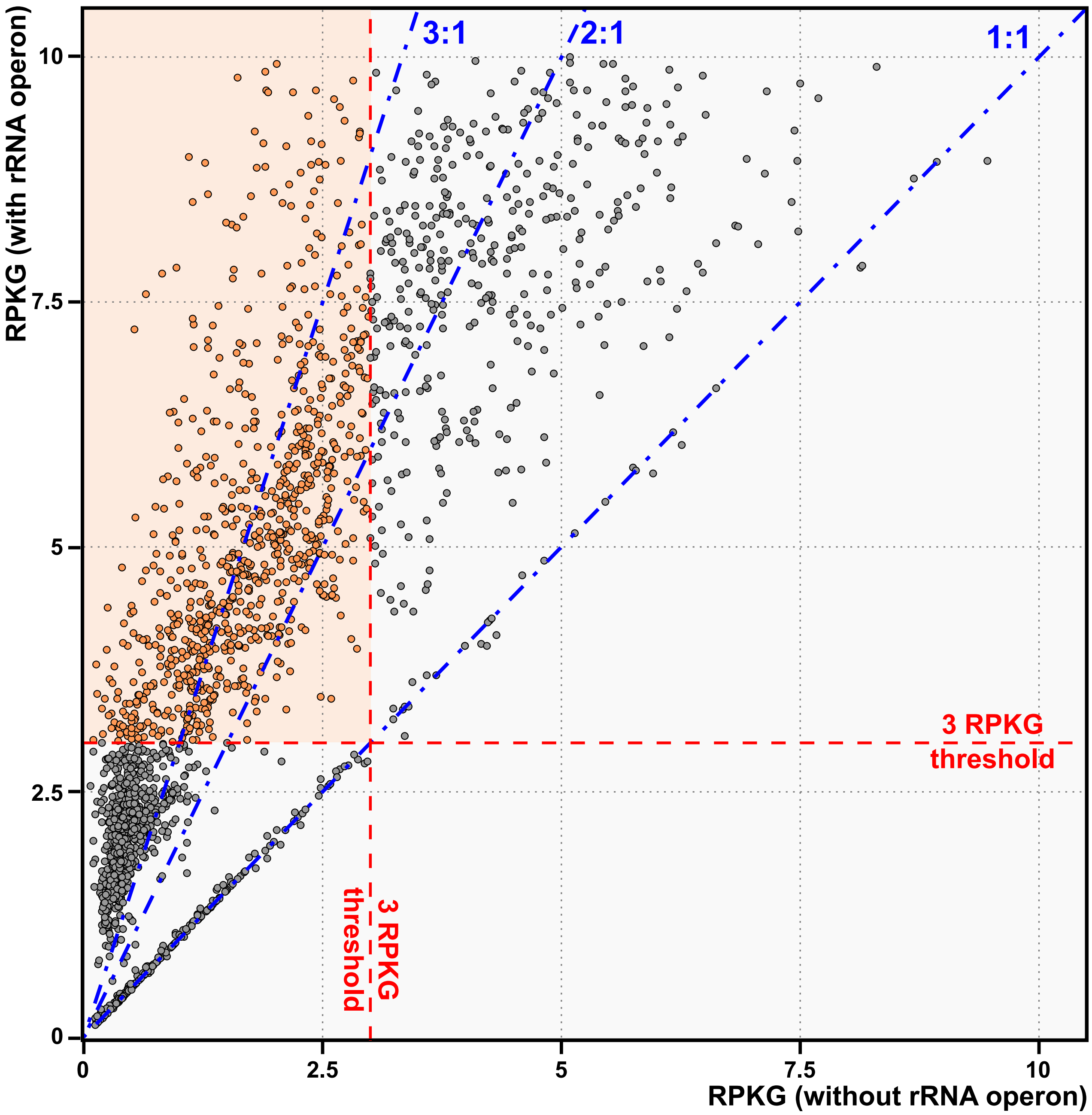
RPKG (with rRNA operon)
RPKG (without rRNA operon)
0
2.5
5
7.5
10
3:1
2:1
1:1
3 RPKG threshold
3 RPKG threshold

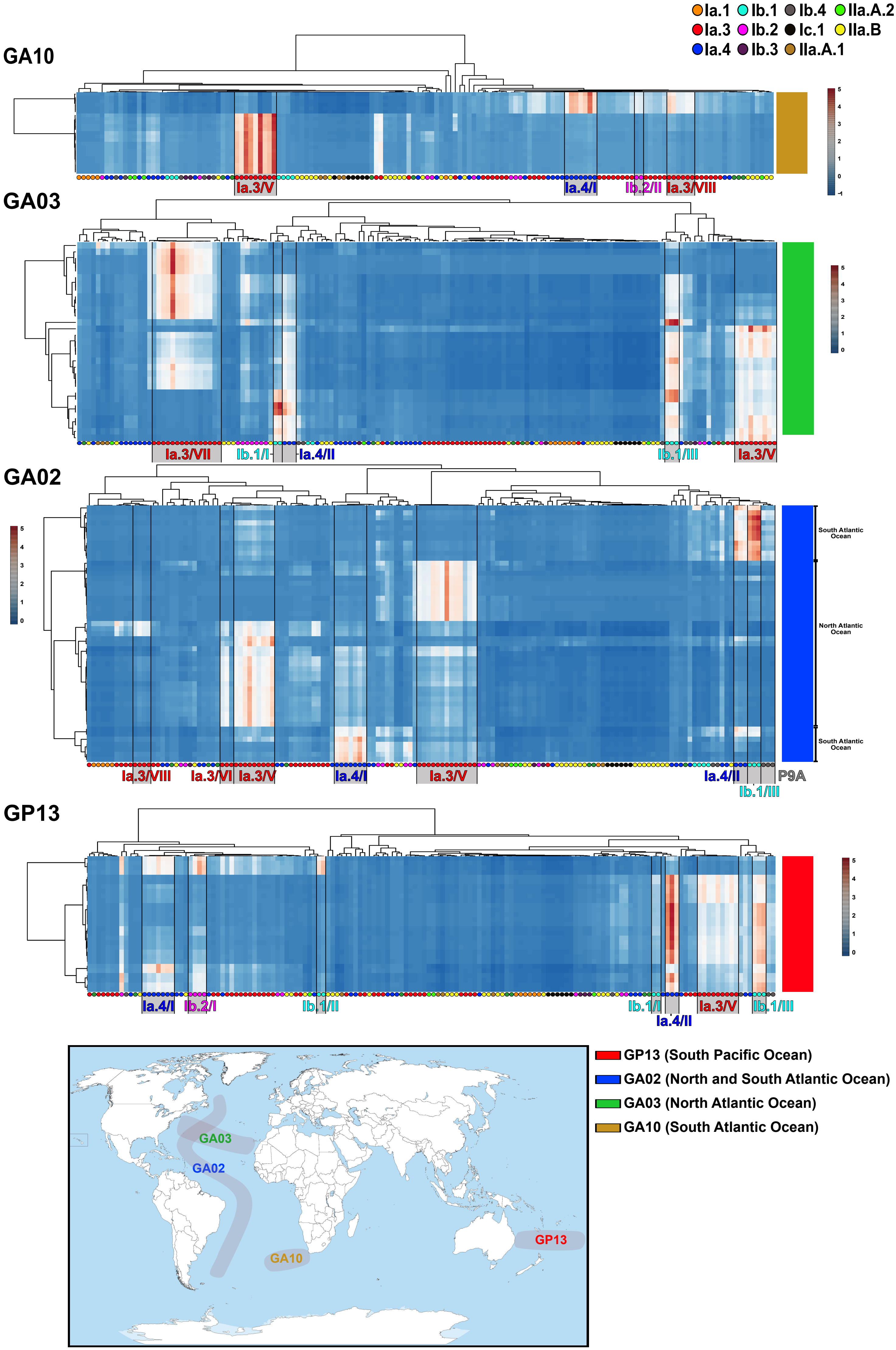
Ia.1 Ib.1 Ib.4 IIa.A.2
Ia.3 Ib.2 Ic.1 IIa.B
Ia.4 Ib.3 IIa.A.1
GA10
Ia.3/V
Ia.4/I
Ib.2/II
Ia.3/VIII
GA03
Ia.3/VII
Ib.1/I
Ia.4/II
Ib.1/III
Ia.3/V
GA02
South Atlantic Ocean
North Atlantic Ocean
South Atlantic Ocean
Ia.3/VIII
Ia.3/VI
Ia.3/V
Ia.4/I
Ia.3/V
Ia.4/II
Ib.1/III
P9A
GP13
Ia.4/I
Ib.2/I
Ib.1/II
Ib.1/I
Ia.4/II
Ia.3/V
Ib.1/III
GA03
GA02
GA10
GP13
GP13 (South Pacific Ocean)
GA02 (North and South Atlantic Ocean)
GA03 (North Atlantic Ocean)
GA10 (South Atlantic Ocean)

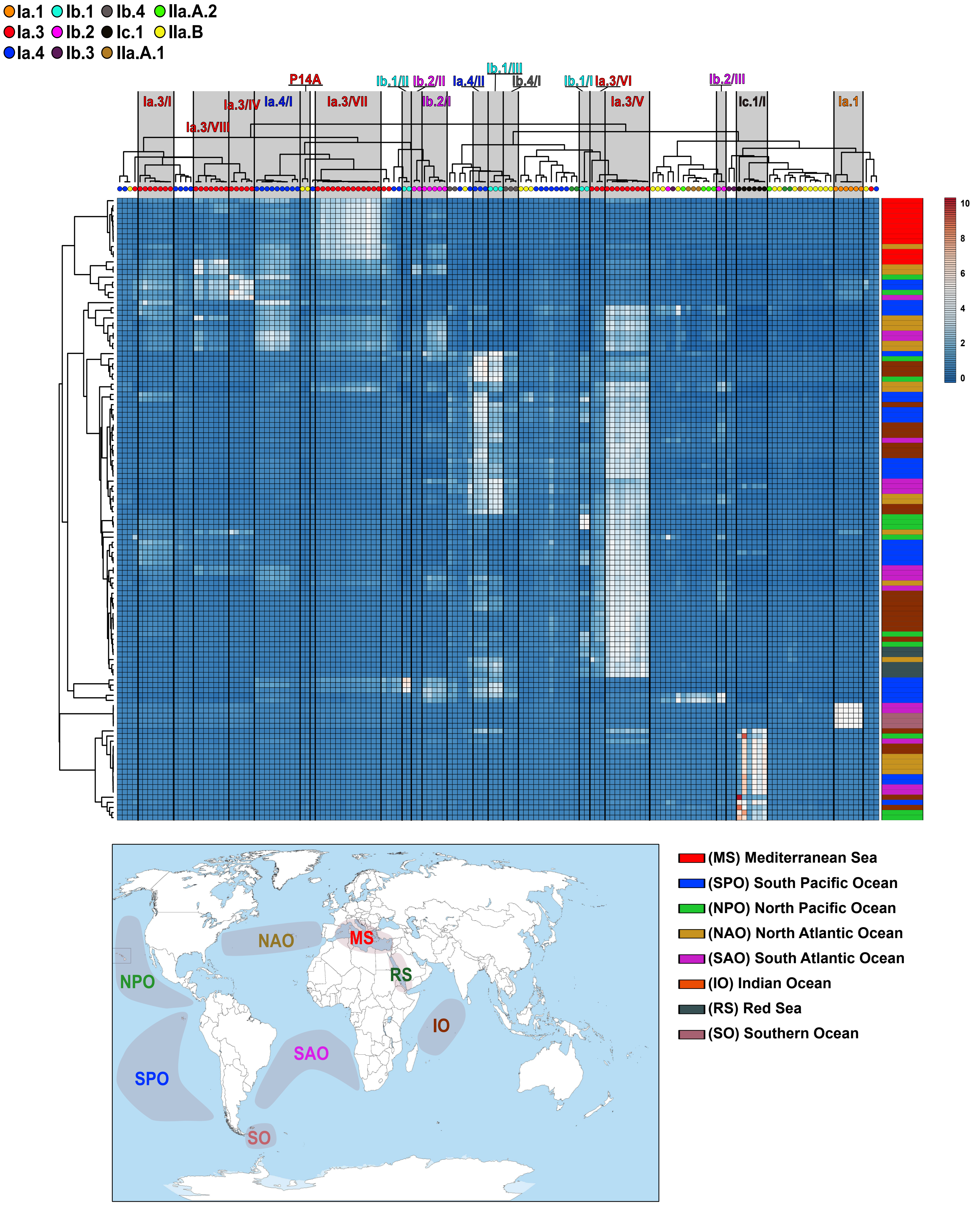
Ia.1
Ib.1
Ib.4
IIa.A.2
Ia.3
Ib.2
Ic.1
IIa.B
Ia.4
Ib.3
IIa.A.1
P14A
Ib.1/III
Ib.1/II
Ib.2/II
Ia.4/II
Ib.4/I
Ib.1/I
Ia.3/VI
Ib.2/III
Ia.3/I
Ia.3/IV
Ia.4/I
Ia.3/VII
Ib.2/I
Ia.3/V
Ic.1/I
Ia.1
Ia.3/VIII
10
8
6
4
2
0
NAO
MS
NPO
RS
IO
SAO
SPO
SO
(MS) Mediterranean Sea
(SPO) South Pacific Ocean
(NPO) North Pacific Ocean
(NAO) North Atlantic Ocean
(SAO) South Atlantic Ocean
(IO) Indian Ocean
(RS) Red Sea
(SO) Southern Ocean

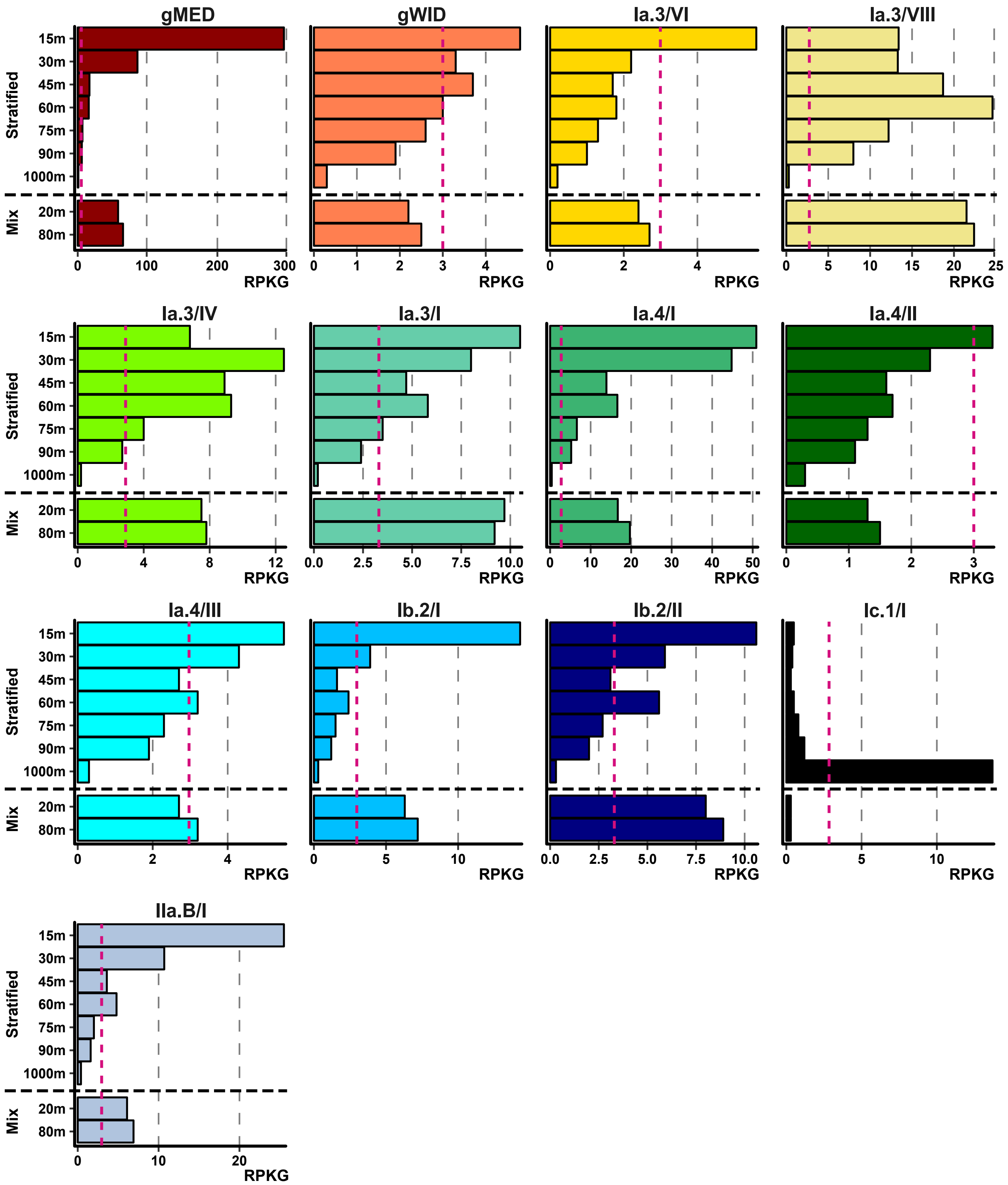
gMED
gWID
Ia.3/VI
Ia.3/VIII
Ia.3/IV
Ia.3/I
Ia.4/I
Ia.4/II
Ia.4/III
Ib.2/I
Ib.2/II
Ic.1/I
IIa.B/I
Stratified
Mix
15m
30m
45m
60m
75m
90m
1000m
20m
80m
RPKG
0 100 200 300
0 1 2 3 4
0 2 4
0 5 10 15 20 25
0 4 8 12
0.0 2.5 5.0 7.5 10.0
0 10 20 30 40 50
0 1 2 3
0 2 4
0 5 10
0.0 2.5 5.0 7.5 10.0
0 5 10
0 10 20

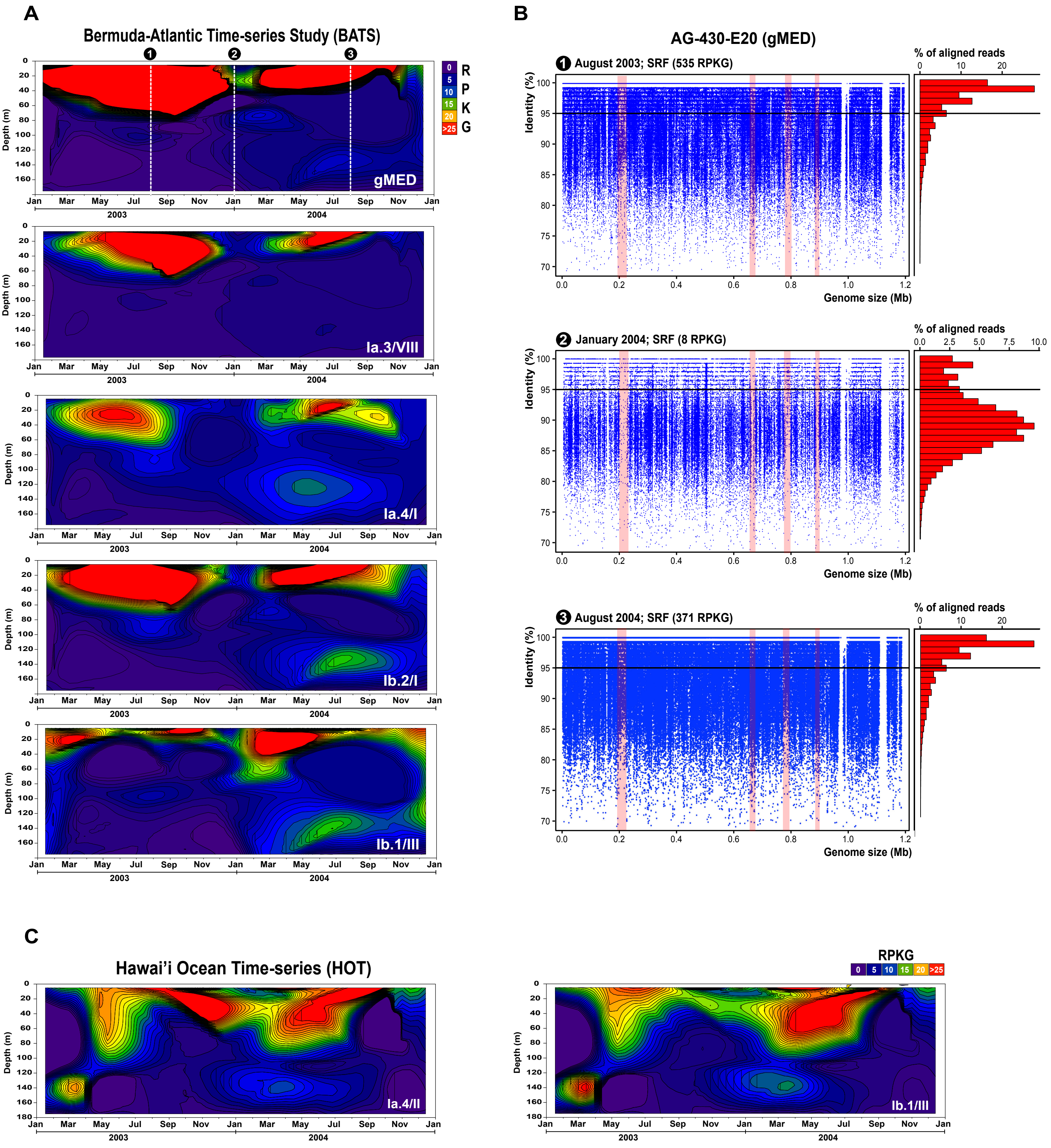
A
Bermuda-Atlantic Time-series Study (BATS)
Depth (m)
gMED
Ia.3/VIII
Ia.4/I
Ib.2/I
Ib.1/III
Jan Mar May Jul Sep Nov Jan Mar May Jul Sep Nov Jan
2003
2004
0 5 10 15 20 >25
R P K G
B
AG-430-E20 (gMED)
August 2003; SRF (535 RPKG)
January 2004; SRF (8 RPKG)
August 2004; SRF (371 RPKG)
Identity (%)
Genome size (Mb)
% of aligned reads
C
Hawai'i Ocean Time-series (HOT)
Ia.4/II
Ib.1/III
RPKG

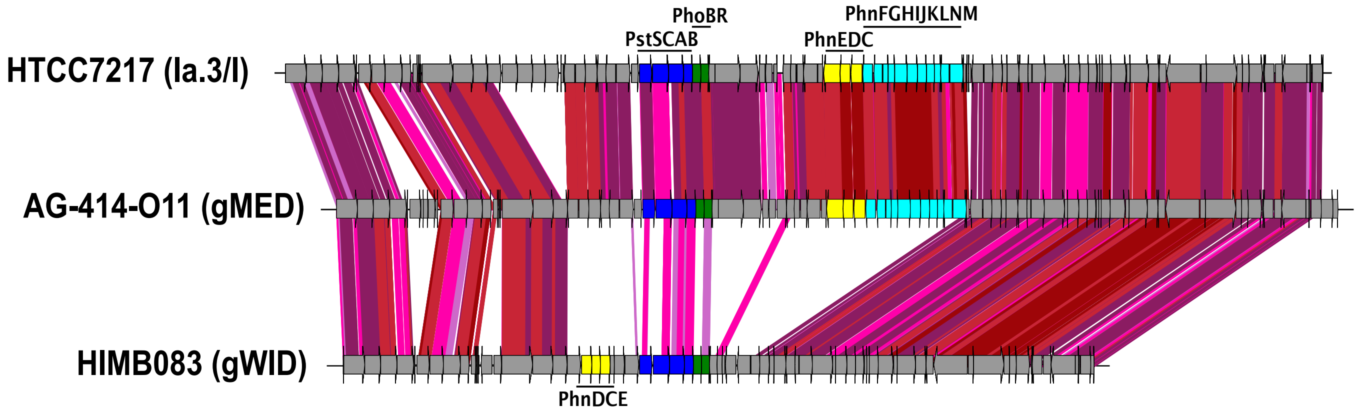
PhoBR
PhnFGHIJKLNM
PstSCAB
PhnEDC
HTCC7217 (Ia.3/I)
AG-414-O11 (gMED)
HIMB083 (gWID)
PhnDCE

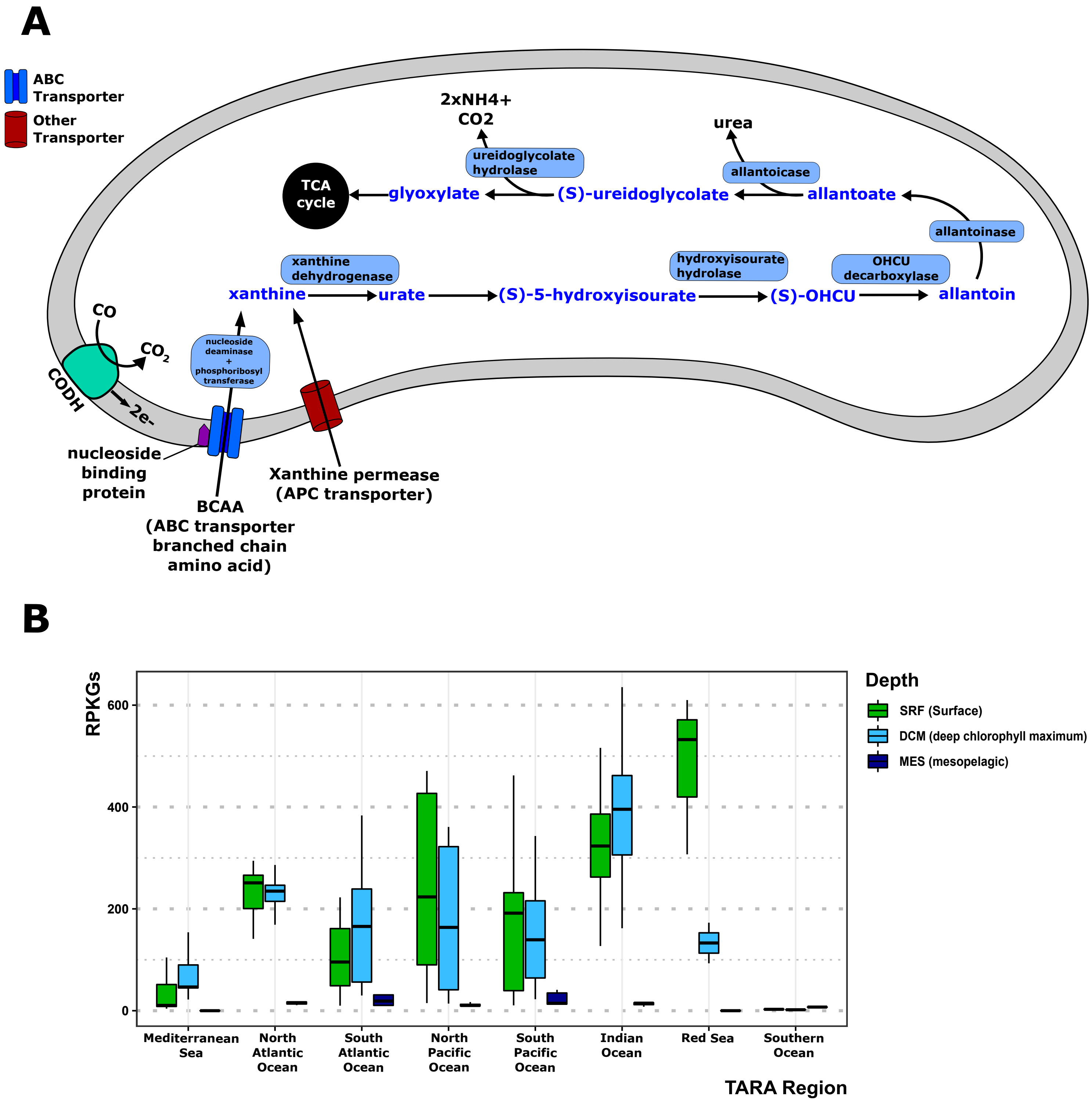

A
ABC Transporter
Other Transporter
2xNH4+
CO2
urea
ureidoglycolate hydrolase
allantoicase
TCA cycle
glyoxylate
(S)-ureidoglycolate
allantoate
allantoinase
xanthine dehydrogenase
hydroxyisourate hydrolase
OHCU decarboxylase
xanthine
urate
(S)-5-hydroxyisourate
(S)-OHCU
allantoin
CO
CO2
CODH
2e-
nucleoside deaminase + phosphoribosyl transferase
nucleoside binding protein
BCAA (ABC transporter branched chain amino acid)
Xanthine permease (APC transporter)
B
RPKGs
600
400
200
0
Mediterranean Sea
North Atlantic Ocean
South Atlantic Ocean
North Pacific Ocean
South Pacific Ocean
Indian Ocean
Red Sea
Southern Ocean
TARA Region
Depth
SRF (Surface)
DCM (deep chlorophyll maximum)
MES (mesopelagic)

A

	HOT162 (NPO-SRF)	TARA_102 (SPO-SRF)	TARA_137 (NPO-SRF)	TARA_031 (RS-SRF)	TARA_036 (IO-SRF)	TARA_038 (IO-SRF)	TARA_034 (RS-SRF)	TARA_033 (RS-SRF)
HOT162 (NPO-SRF)	1							
TARA_102 (SPO-SRF)	-0.05	1						
TARA_137 (NPO-SRF)	-0.04	0.49	1					
TARA_031 (RS-SRF)	0.12	0.11	0.19	1				
TARA_036 (IO-SRF)	-0.03	0.23	0.29	0.35	1			
TARA_038 (IO-SRF)	-0.02	0.23	0.3	0.39	0.76	1		
TARA_034 (RS-SRF)	-0.02	0.1	0.15	0.39	0.58	0.59	1	
TARA_033 (RS-SRF)	-0.05	0.06	0.14	0.38	0.52	0.52	0.72	1

gWID (HIMB083)
Horizontal microdiversity

B

	SRR2103014 (RS-10m)	SRR2103015 (RS-25m)	SRR2103016 (RS-50m)	SRR2103018 (RS-100m)
SRR2103014 (RS-10m)	1			
SRR2103015 (RS-25m)	0.02	1		
SRR2103016 (RS-50m)	-0.04	0.04	1	
SRR2103018 (RS-100m)	0.00	0.02	0.01	1

gWID (HIMB083)
Vertical microdiversity

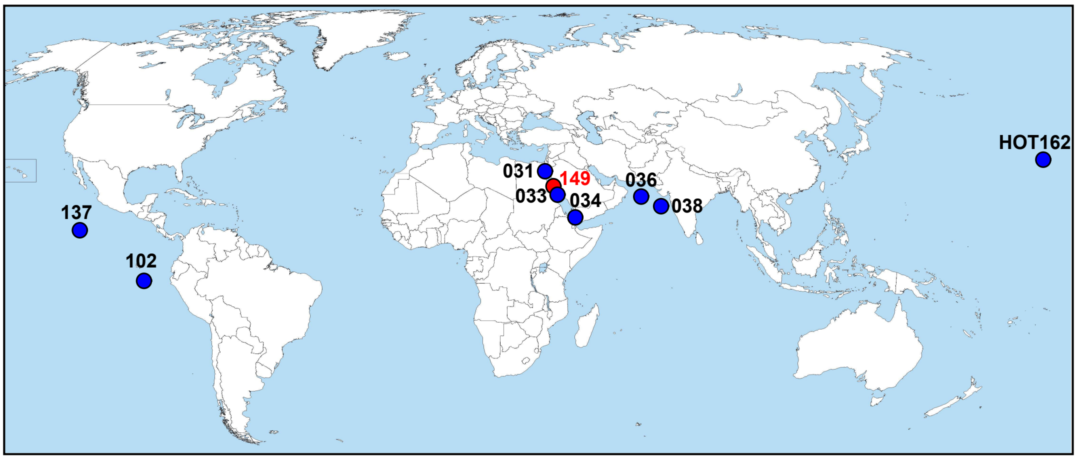

Supplement: Supplementary file 1 — Fig. S1: Neighbour‐joining phylogenetic tree (145 sequences, 1000 bootstraps, Jukes–Cantor distance correction) of the internal space transcriber (ITS) located between the 16S and the 23S rRNA operon. Nomenclature for phylotype assignments is derived from Brown and colleagues (2012), Ngugi and Stingl (2012) and Jimenez‐Infante and colleagues (2017). Reference genomes, reference ITS sequences and genomes analysed in this study are shown in blue, green and red, respectively. Fig. S2: Pairwise comparison among the SAR11 genomes using both amino acid identity (AAI) and average nucleotide identity (ANI). Rectangles with continuous and dotted line delimit subclades and genomospecies, respectively. Fig. S3: Pairwise comparison among SAGs and isolated reference genomes of the SAR11 IV and V subclades. Genomes of the Pelagibacterales order (clades Ia to IIIa) were included in the analysis and are highlighted with a green rectangle. A) Average nucleotide identity (ANI) distance matrix. B) Percentage of the genome aligned (coverage) during the ANI analysis. C) Average amino acid identity (AAI) distance matrix. D) Percentage of proteins shared during the AAI analysis. Fig. S4: Comparison of the abundance of 185 SAR11 genomes (we excluded the clades IV and V, whose affiliation to the SAR11 clade is controversial) in 20 randomly selected TARA metagenomes. RPKG values obtained after the removal of the ribosomal RNA operon (x axis) were compared to those obtained recruiting the whole genome (y axis). Only reads recruiting >98% identity with an alignment >50 bp long were considered. Dashed red lines represent the threshold of 3 RPKG applied to discriminate between presence (>3) or absence (<3) of a genome in a sample. Dashed blue lines indicate the ratio between RPKG values. The area framed in orange includes all those genomes which, if the ribosomal operon had not been eliminated, would have given a false positive (ca. 32.5%). Fig. S5: Clustering of the SAR11 genomes recruited alon [file EMI-22-1748-s001.pdf]
